# Correction: Inactivation of branched-chain amino acid uptake halts *Staphylococcus aureus* growth and induces bacterial quiescence within macrophages

**DOI:** 10.1371/journal.ppat.1014036

**Published:** 2026-03-10

**Authors:** Adriana Moldovan, Ronald S. Flannagan, Marcel Rühling, Kathrin Stelzner, Clara Hans, Kerstin Paprotka, Tobias C. Kunz, David E. Heinrichs, Thomas Rudel, Martin J. Fraunholz

S13 Fig was uploaded incorrectly. Please see the correct S13 Fig here.

## Supporting information

S13 Fig Valine and leucine rescue the growth defect of *brnQ1*- deficient *S. aureus* in macrophages. (a) *S. aureus* JE2 growth in a chemically defined media, either containing 1 mM of each BCAA (CDM) or lacking the stated amino acid(s). OD_600_ was measured every 18 min, for 48h. Data are shown as mean values ±SD of independent experiments (n = 4). (b) Intracellular growth of a *S. aureus brnQ1* in RAW 264.7 macrophages. Macrophages were infected with either WT USA300 or *S. aureus brnQ1* and, after gentamicin treatment, supplemented with either valine (Val), leucine (Leu), or Leu and Val (each at 1 mM final concentration) for the duration of the experiment. Data are shown as the mean log10 value ± S.D. for the calculated fold change in CFU/mL at 20h relative to 1.5h *p.i.*, for each bacterial strain. Each data point plotted represents a biological replicate derived from at least three independent experiments (n ≥ 3). Statistical analysis: Brown-Forsythe and Welch ANOVA with Dunnett’s T3 multiple comparison test. (c) Fluorescence microscopy micrographs of RAW 264.7 macrophages infected with *S. aureus* USA300 WT and *S. aureus brnQ1* mutant expressing GFP (green) that were labeled with a fluorescent proliferation dye (blue). The macrophage plasmalemma and extracellular cocci are stained with TMR-WGA (red). 1 mM Val and Leu (each) were added to the medium after gentamicin treatment (1.5h *p.i.*). At the outset (i.e., 1.5h) all bacteria are GFP and proliferation dye positive; over time and with replication, GFP-positive yet proliferation dye negative bacteria that are devoid of WGA can be seen at 10h and 20h *p.i*. White arrows indicate unrestricted bacterial replication (*URG*). Shown are representative micrographs, from 2 independent experiments (n = 2) (scale bars= ~ 10 µm).
(TIF)
