# Supplementary figures and images for: Inactivation of branched-chain amino acid uptake halts Staphylococcus aureus growth and induces bacterial quiescence within macrophages
Source: PLoS Pathog. 2025 Aug 8;21(8):e1013291. doi: 10.1371/journal.ppat.1013291 (PMC12333996; doi:10.1371/journal.ppat.1013291)

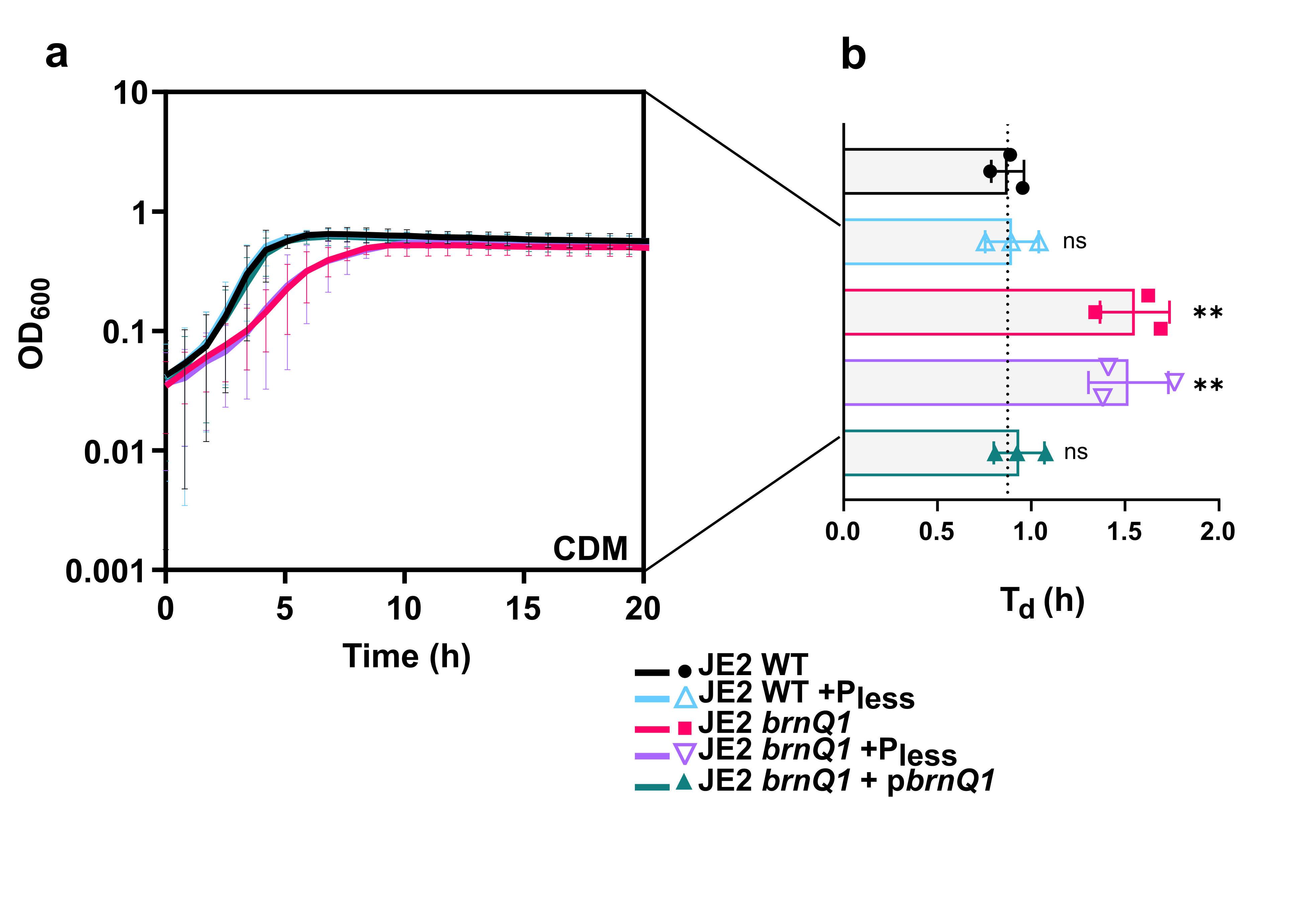

Supplement: S1 Fig — A functional copy of the brnQ1 gene, under its native promoter region, (SAUSA300_0188/ SAUSA300_RS00985) was cloned into a high-copy plasmid and used for complementing JE2 brnQ1 to yield JE2 brnQ1 + pbrnQ1. To exclude potential negative effects on bacterial fitness caused by gene overexpression from a high-copy plasmid, constructs carrying the brnQ1 gene without promoter sequence were created (termed “Pless”). (a) Effect of the complementation plasmid (pbrnQ1) or promoter-less brnQ1 plasmid (Pless) on bacterial growth in a chemically defined medium with 1 mM of each BCAA (CDM). OD600 was determined every 18 min, for 20h. Data are shown as mean values from independent experiments ± SD (n = 3). (b) Doubling times (Td) estimated from the growth curve. Dotted line corresponds to mean Td for JE2 WT. Statistical analysis: one-way ANOVA, with Dunnett’s multiple comparisons (vs JE2 WT); ns = not significant; **p < 0.01. (TIF) [file ppat.1013291.s001.tif]

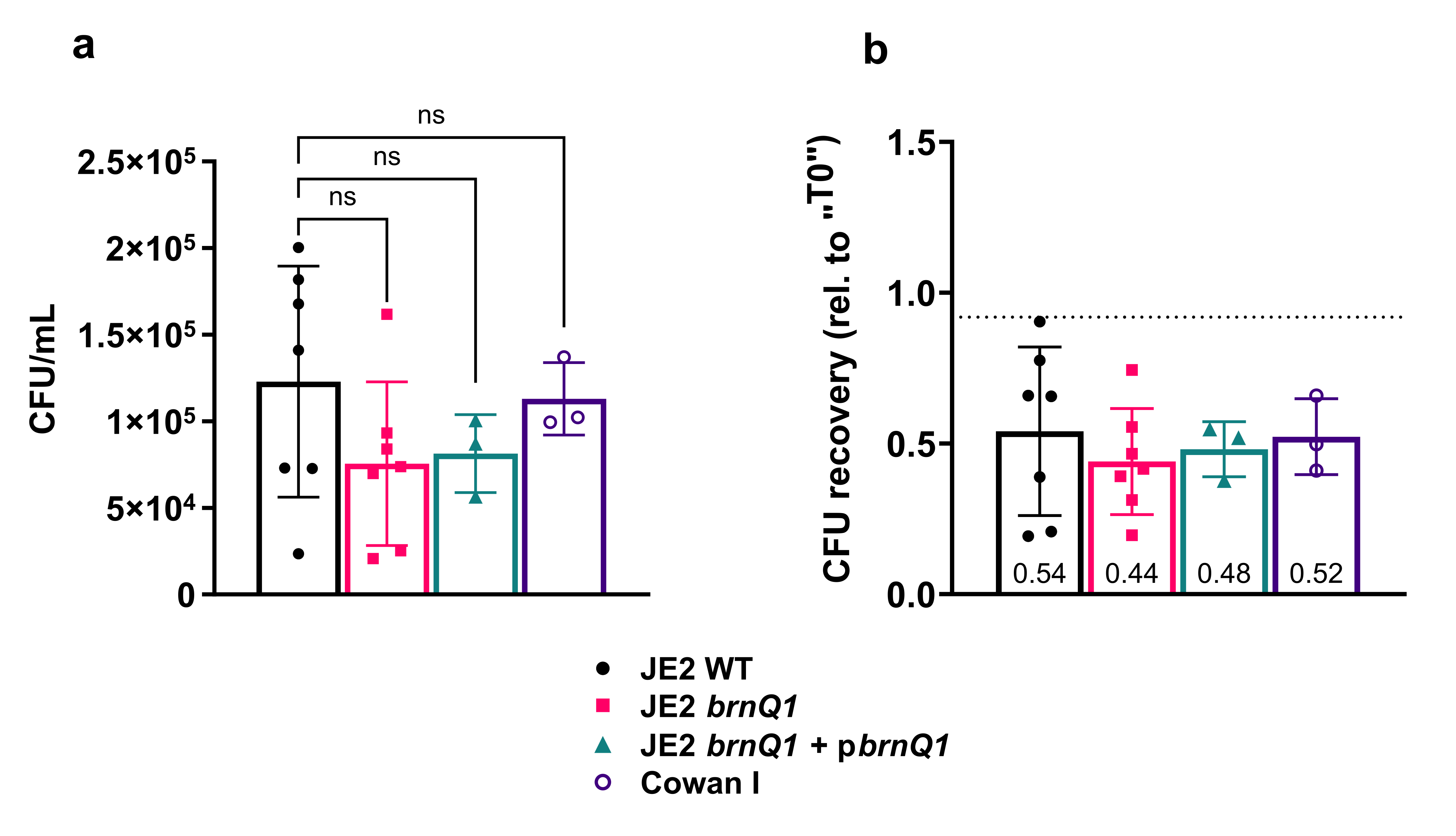

Supplement: S2 Fig — (a) Bacteria recovered from infected macrophages at 6h p.i. a Raw CFU counts (CFU/mL) plotted from data set shown in Fig 1d. Data are shown as mean ±SD from independent experiments (JE2 WT and JE2 brnQ1: n = 7, JE2 brnQ1 + pbrnQ1 and Cowan I: n = 3). (b) CFU counts shown in a, normalised to “T0” (i.e., 45 min p.i.). Numbers inside bars represent the plotted value (mean). Statistical analysis (a): one-way ANOVA, with Dunnett’s multiple comparisons, vs JE2 WT; ns = not significant. (TIF) [file ppat.1013291.s002.tif]

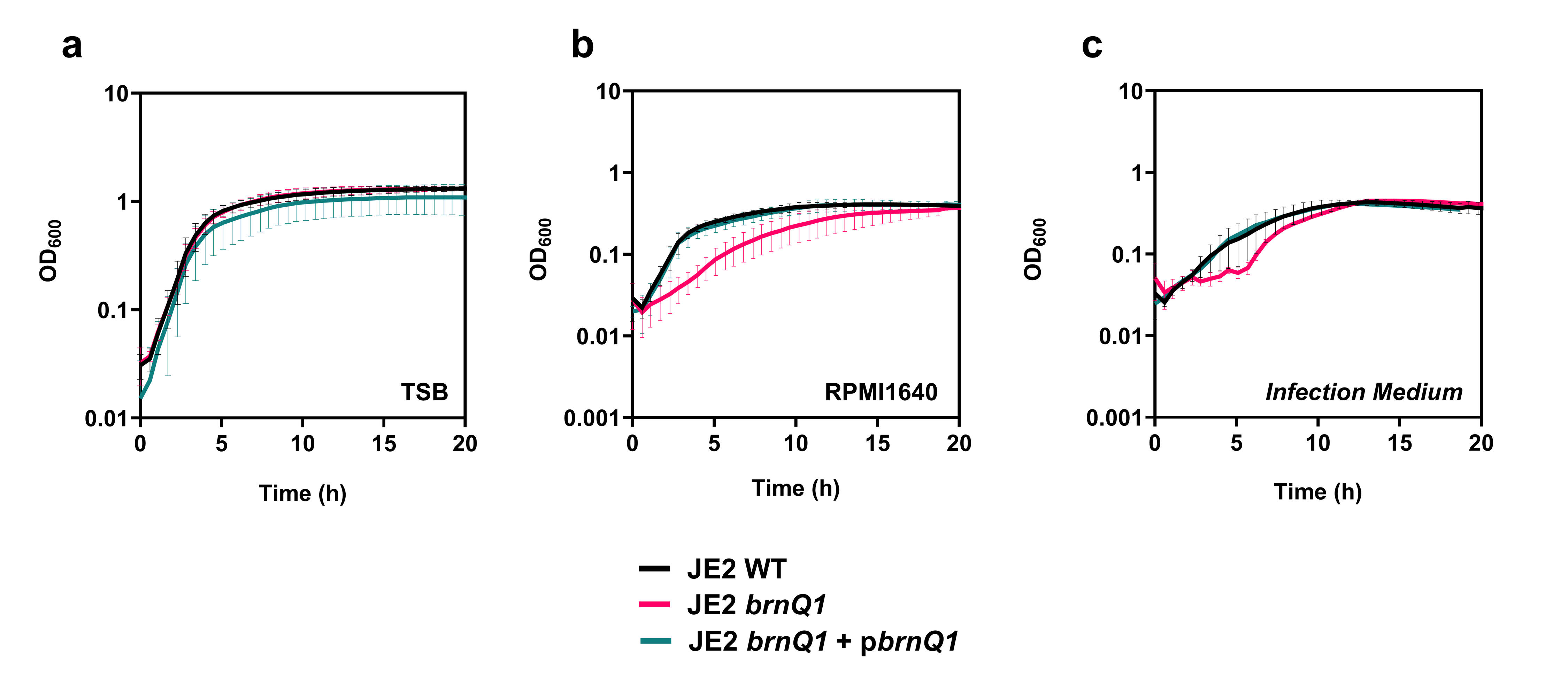

Supplement: S3 Fig — S. aureus growth in (a) the nutrient-rich tryptic soy broth medium (TSB), (b) RPMI1640 and (c) Infection Medium (RPMI1640 + 10% v/v heat inactivated FBS). OD600 was measured every 18 min, for 20h. Data are shown as mean values ± SD from independent experiments: (a) JE2 WT and JE2 brnQ1: n = 5, JE2 brnQ1 + pbrnQ1: n = 2; (b) JE2 WT and JE2 brnQ1: n = 6, JE2 brnQ1 + pbrnQ1: n = 4; (c) JE2 WT and JE2 brnQ1: n = 3, JE2 brnQ1 + pbrnQ1: n = 1. (TIF) [file ppat.1013291.s003.tif]

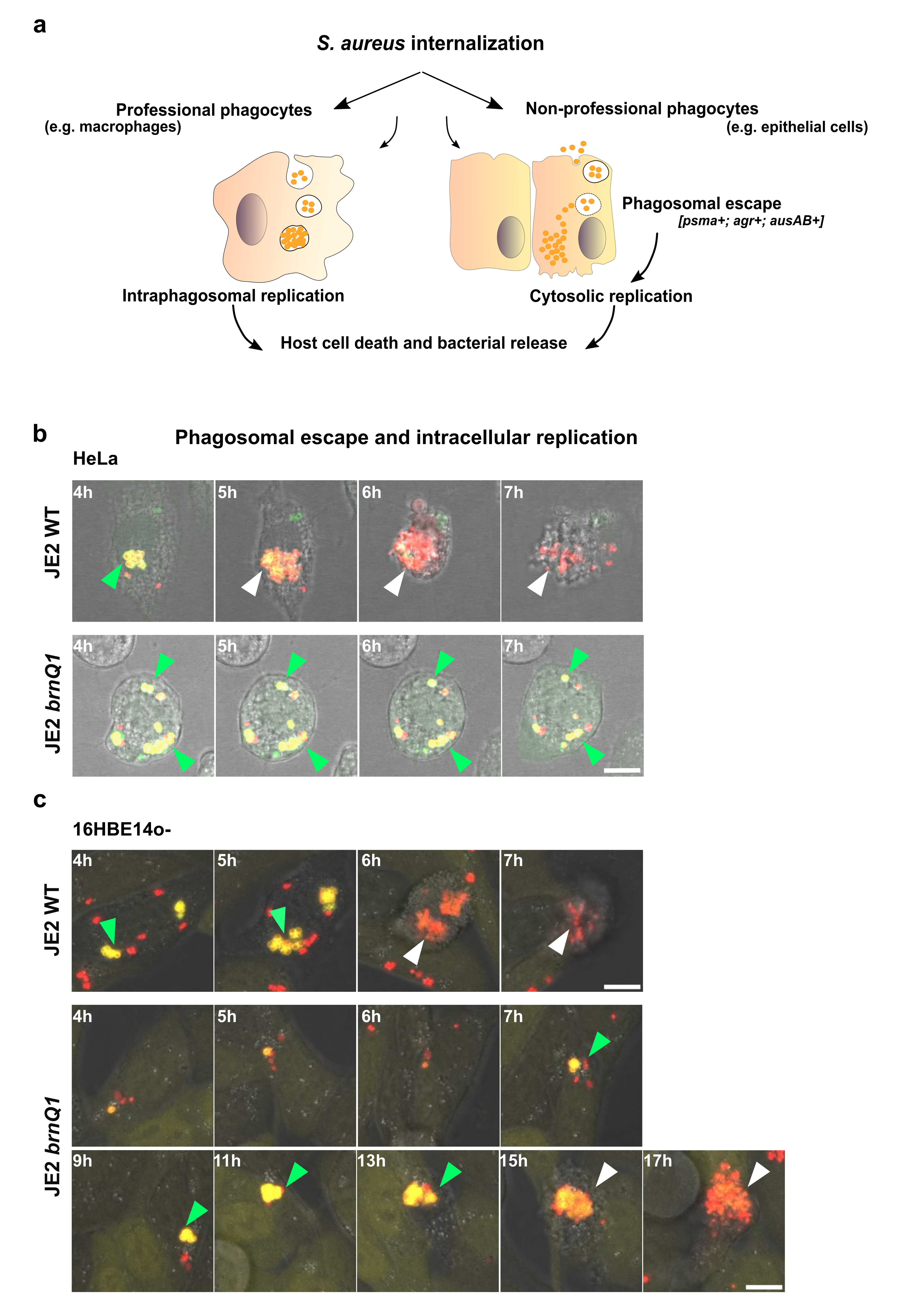

Supplement: S4 Fig — (a) Dynamics of S. aureus infection in professional phagocytes (e.g., macrophages) vs non-professional phagocytes (e.g., epithelial or endothelial cells). Phenol-soluble modulins (psm), the agr quorum sensing system (agr) and the non-ribosomal peptide synthetase AusAB (ausAB) were reported as bacterial factors involved in the phagosomal escape of S. aureus (modified after [7]). Live cell imaging of reporter (b) HeLa cells or (c) human bronchial epithelial cells 16HBE14o- constitutively expressing the phagosomal escape marker YFP-CWT, were infected with mRFP-expressing JE2 WT or JE2 brnQ1. Green arrows indicate phagosomal escape events and white arrows indicate intracellular bacterial replication. Shown are micrographs extracted from time-lapse series, representative of 3 independent experiments (n = 3). Scale bars = 10 µm (see also S2–S5 Movies). (16HBE14o-: immortalized human bronchial epithelial cells). (TIF) [file ppat.1013291.s004.tif]

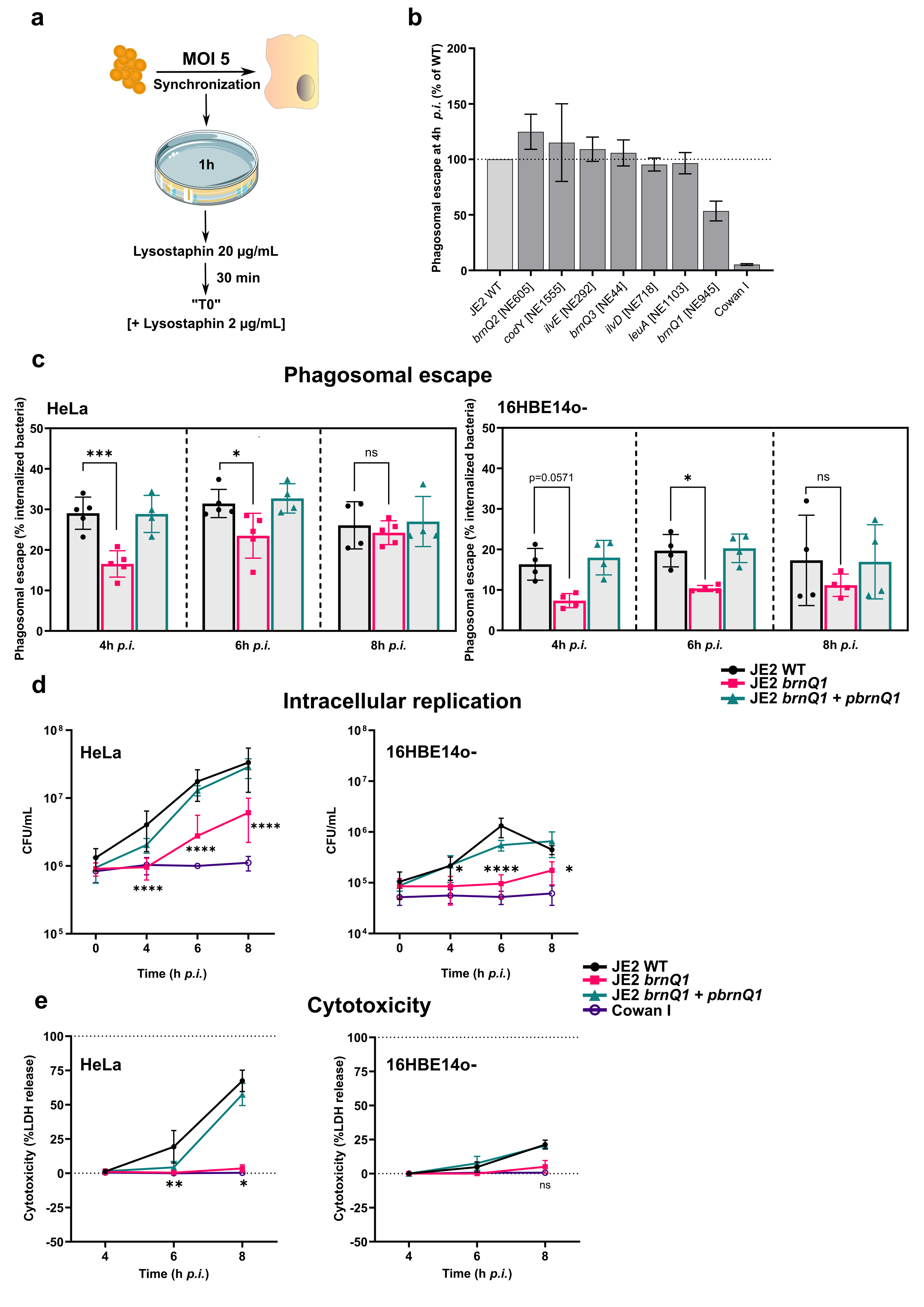

Supplement: S5 Fig — (a) Experimental procedure for epithelial cell infections: cells were infected with S. aureus at a multiplicity of infection (MOI) of 5 for 1h, following synchronization by centrifugation. Extracellular S. aureus were removed by a 30 min treatment with 20 µg/µL lysostaphin. Infected cells were further incubated in the presence of 2 µg/mL lysostaphin. “T0” corresponds to 1.5h p.i. (b) Phagosomal escape rates of S. aureus JE2 WT and Nebraska Transposon Mutant Library (NTML) S. aureus mutants within several genes associated with either BCAA-uptake (brnQ1-3 and bcaP) or biosynthesis (ilvE, ilvD, leuA) were assessed by automated fluorescence microscopy at 4h p.i., in HeLa YFP-CWT phagosomal escape reporter cells. Phagosomal escape rates of the JE2 WT (percent YFP-CWT positive events of total internalized bacteria) were set to 100% (dotted line). S. aureus Cowan I was used as phagosomal escape-negative control. Data are shown as mean ± SD from independent experiments (n = 2). “NE” designates mutant strain identifier in the NTML. All bacterial strains express the mRFP fluorescent protein. (c) Phagosomal escape was quantified in reporter HeLa or 16HBE14o- cells expressing the YFP-CWT phagosomal escape marker, by automated fluorescence microscopy, at different time-points, using SNARF-stained bacteria. Data represent the percent YFP-CWT positive events (phagosomal escape) of total internalized bacteria. Data are shown as mean ± SD from independent experiments (n > 3). (d) Intracellular replication in HeLa and 16HBE14o- epithelial cells. Data are shown as means ± SD from independent experiments (n > 3). (e) Cytotoxicity of S. aureus infection against HeLa and 16HBE14o- epithelial cells (% LDH release). Data are shown as means ± SD from independent experiments (n > 3). Statistical analysis: (c) two-way ANOVA with Dunnett’s multiple comparisons test (each sample vs JE2 WT, for each time-point); (d) two-way ANOVA with Dunnett’s multiple comparisons test, using log10-transformed [file ppat.1013291.s005.tif]

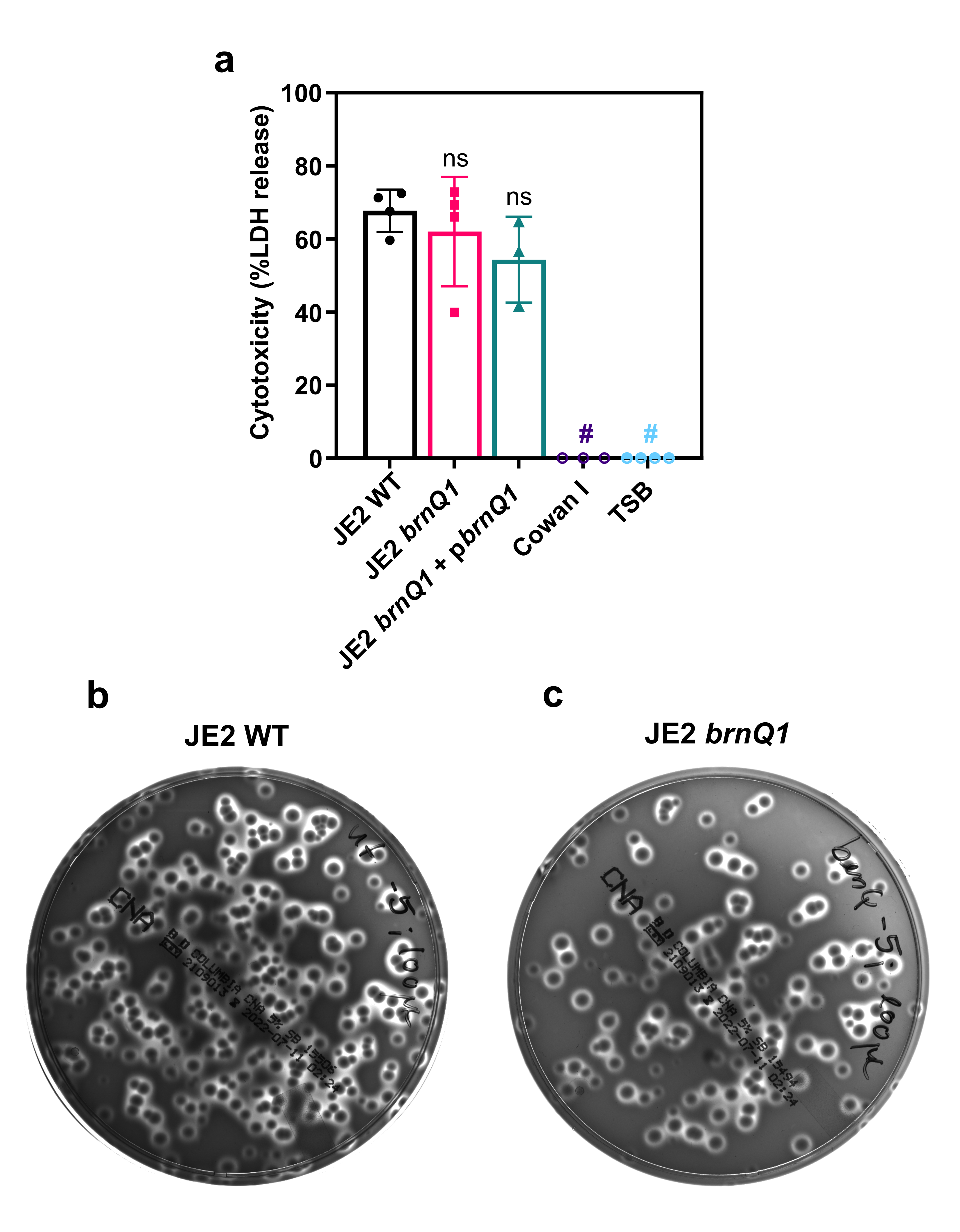

Supplement: S6 Fig — (a) Treatment with 10% sterile stationary phase supernatant (24h, TSB) and its effect on cytotoxicity (% LDH release) against HeLa cells (4h of treatment). S. aureus Cowan I is not cytotoxic against epithelial cells and serves as control. Data are shown as mean values ± SD of independent experiments (JE2 WT and JE2 brnQ1: n = 4, JE2 pbrnQ1 + pbrnQ1 and Cowan I: n = 3). Statistical analysis: one-way ANOVA, with Dunnett’s multiple comparisons test, vs JE2 WT; ns = not significant; #=below detection limit, set to “0”. (b, c) S. aureus JE2 haemolysis on Columbia agar with 5% defibrinated sheep blood, for 30h, at 37°C: (b) JE2 WT and (c) JE2 brnQ1. Representative image is shown (n > 3). (TIF) [file ppat.1013291.s006.tif]

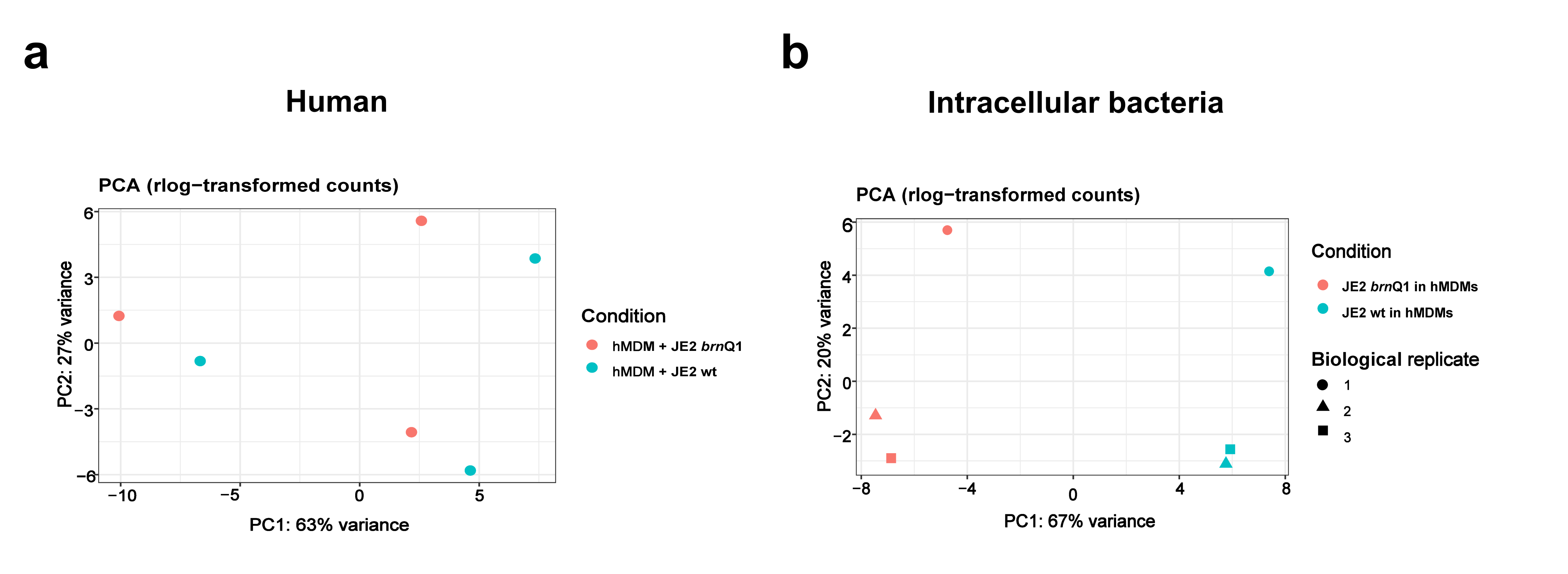

Supplement: S7 Fig — (a) Principal component analysis (PCA) of host transcripts and (b) intracellular bacterial transcripts from dual RNA-seq, from 3 independent experiments (n = 3) (hMDM: primary human monocyte-derived macrophages). (TIF) [file ppat.1013291.s007.tif]

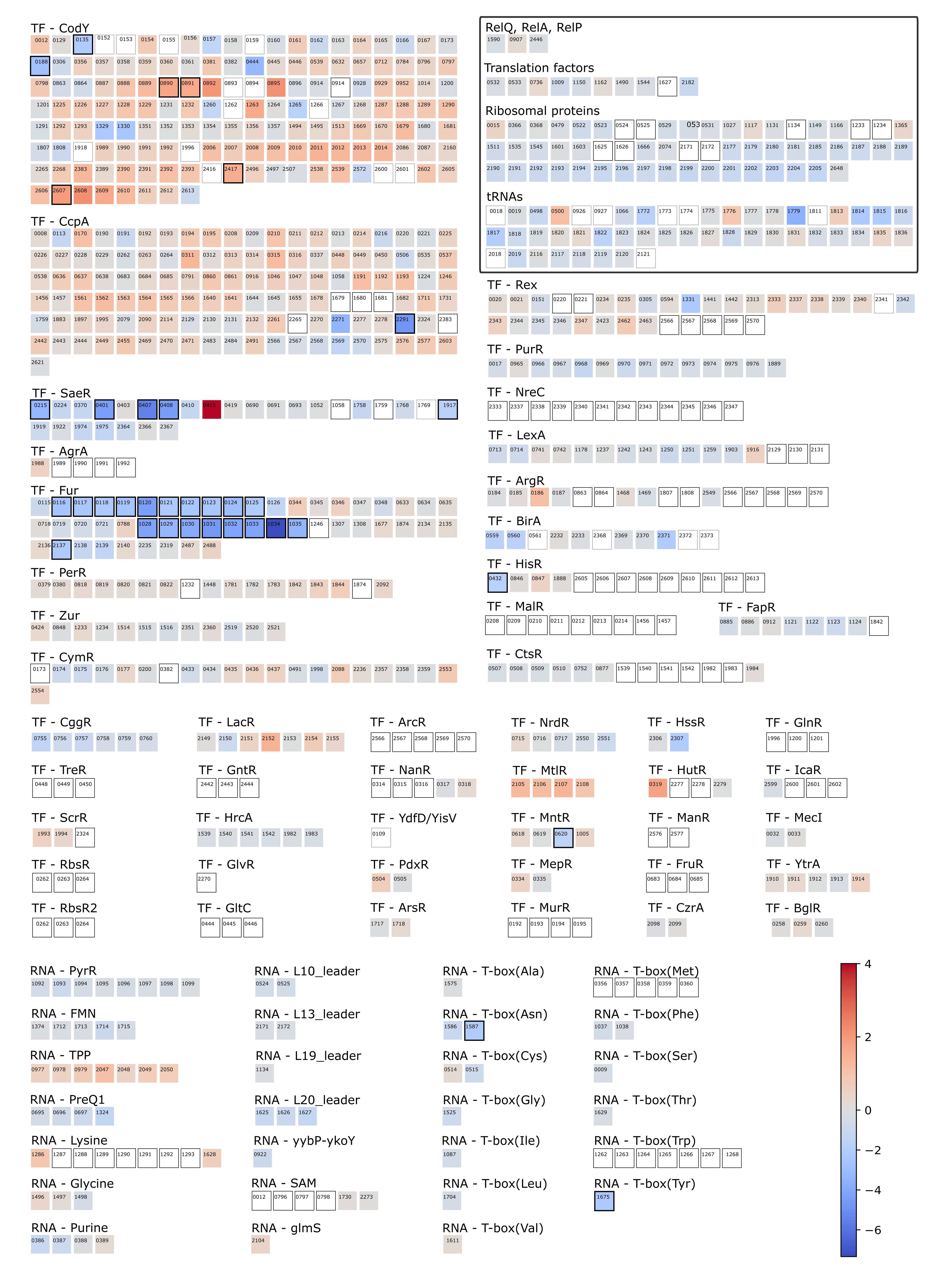

Supplement: S8 Fig — Displayed squares represent a gene of the indicated regulon and box coloration was assigned according to the heat map in the lower right (red: upregulated in JE2 brnQ1 compared to JE2 WT; blue: downregulated in JE2 brnQ1 compared to JE2 WT). Gene-boxes with black frames indicate significance of differential gene expression (padj< 0.05) and absolute values of log2 fold-changes >1.5 or <-1.5. Genes regulated upon bacterial stringent response, such as genes coding for ribosomal proteins or tRNAs, were added to the analysis (framed gene set in the upper right). Gene identifiers correspond to USA300_FPR3757 (old locus ID). Regulons were retrieved from RegPrecise.lbl.gov. (TIF) [file ppat.1013291.s008.tif]

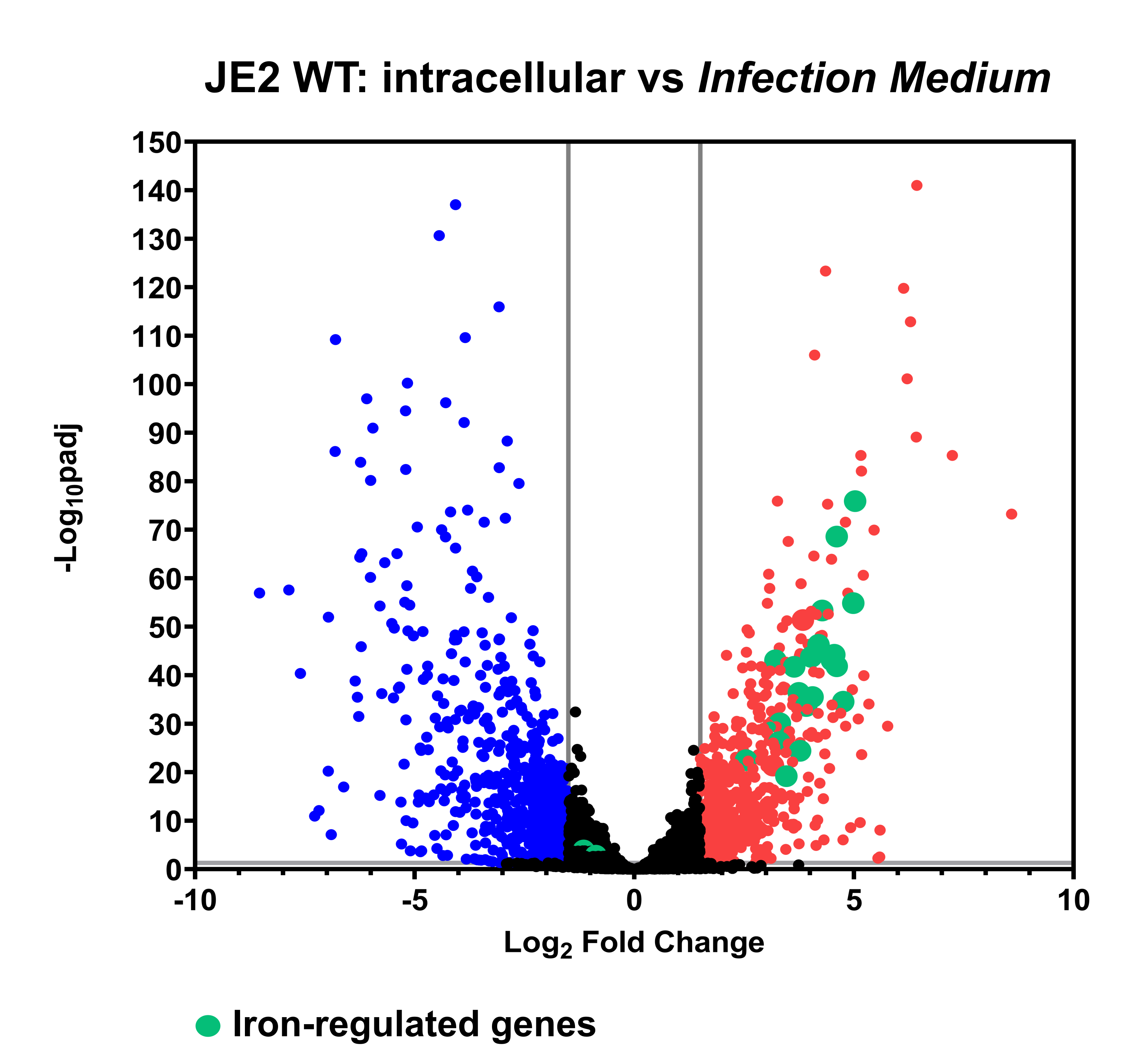

Supplement: S9 Fig — Transcriptomes of intracellular JE2 WT in primary human macrophages at 10h p.i. compared to bacteria grown in Infection Medium for 10h. Horizontal lines represent a p-value cutoff < 0.05. Vertical lines represent cutoffs of log2fold changes of either <-1.5 or > 1.5. Experiment was run in parallel with experiments shown in Fig 2a-b. Data from Fig 2b were used for comparison and expressed as “intracellular vs Infection Medium” using DESeq2. Transcripts of iron uptake systems, which are prominently differentially regulated in intracellular bacteria vs Infection Medium, are depicted in green. Data are shown from 3 independent experiments (n = 3). (TIF) [file ppat.1013291.s009.tif]

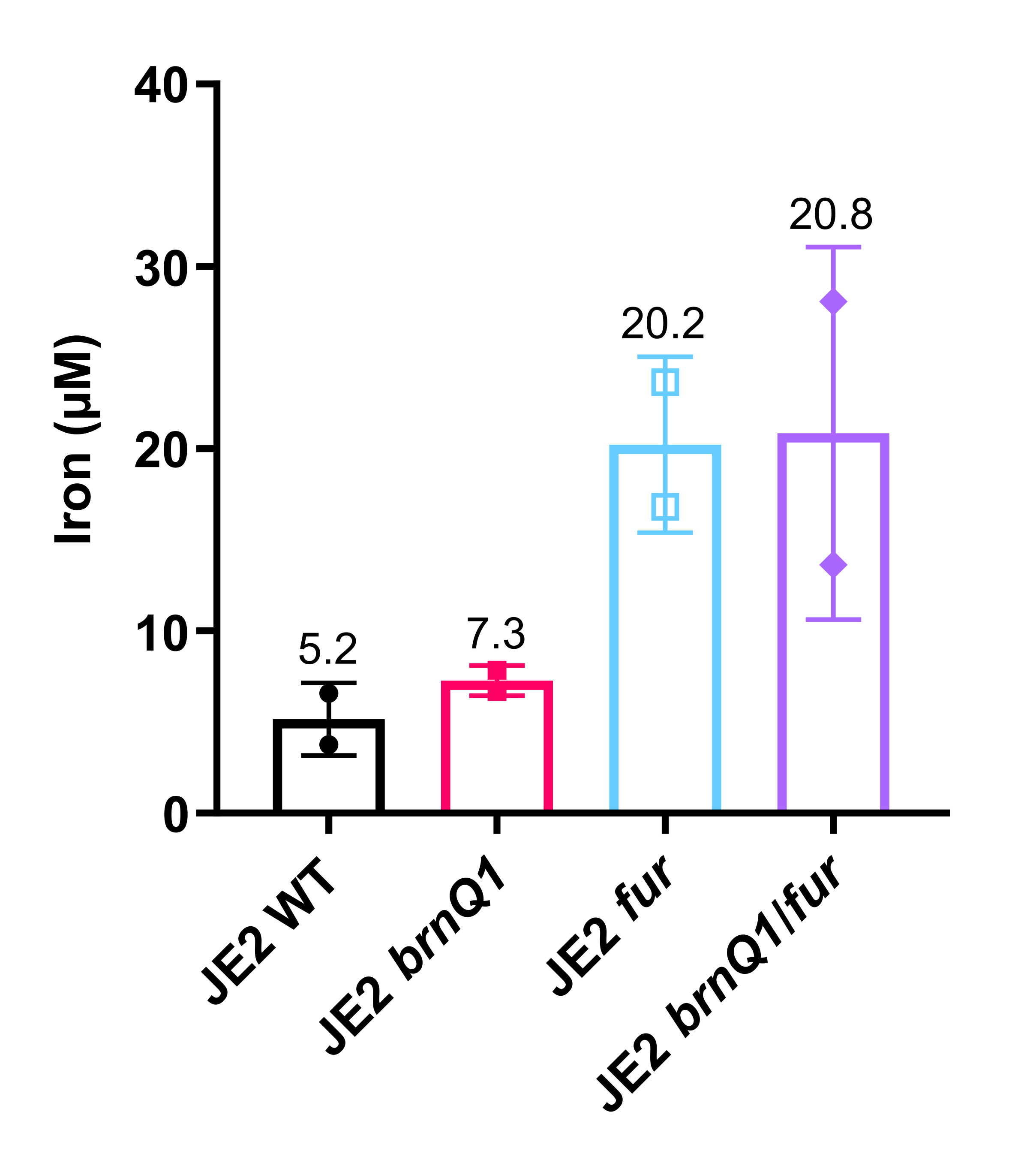

Supplement: S10 Fig — Total iron (Fe2+ and Fe3+) measured in bacteria grown for 24h in RPMI1640 medium supplemented with 20 µM Fe2+ (provided as FeSO4). Data are shown as mean values ±SD of independent experiments (n = 2). (TIF) [file ppat.1013291.s010.tif]

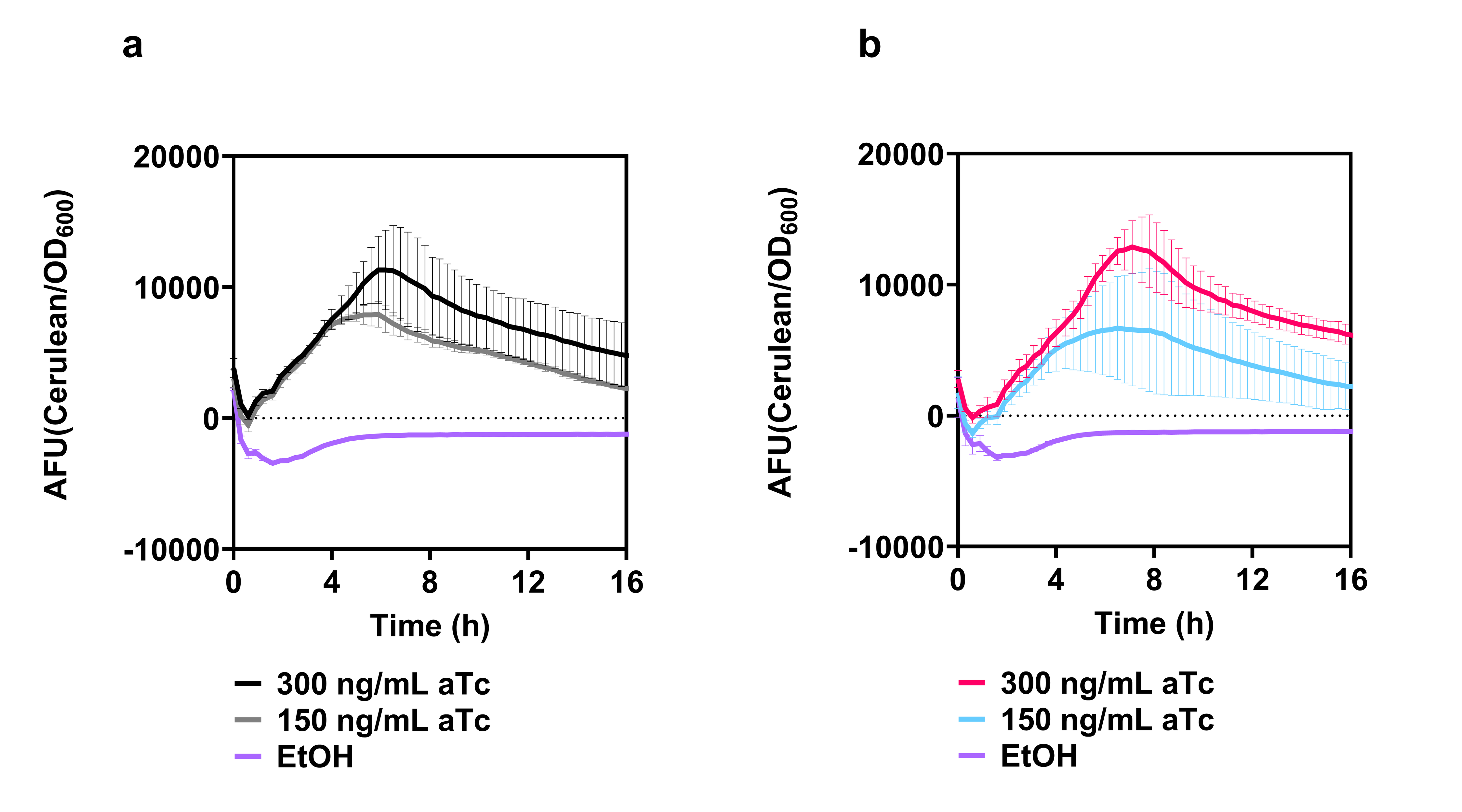

Supplement: S11 Fig — (a) JE2 WT and (b) JE2 brnQ1 were grown in TSB medium supplemented with 300 ng/mL aTc (concentration used in infection experiments), 150 ng/mL or vehicle control (100% ethanol). OD600 and fluorescence (Ex/Em 433/504 nm) were measured every 18 min for 16h. Data are shown as mean values ±SD of independent experiments (n = 3). (AFU = arbitrary fluorescence units, expressed as Cerulean/OD600 ratio). (TIF) [file ppat.1013291.s011.tif]

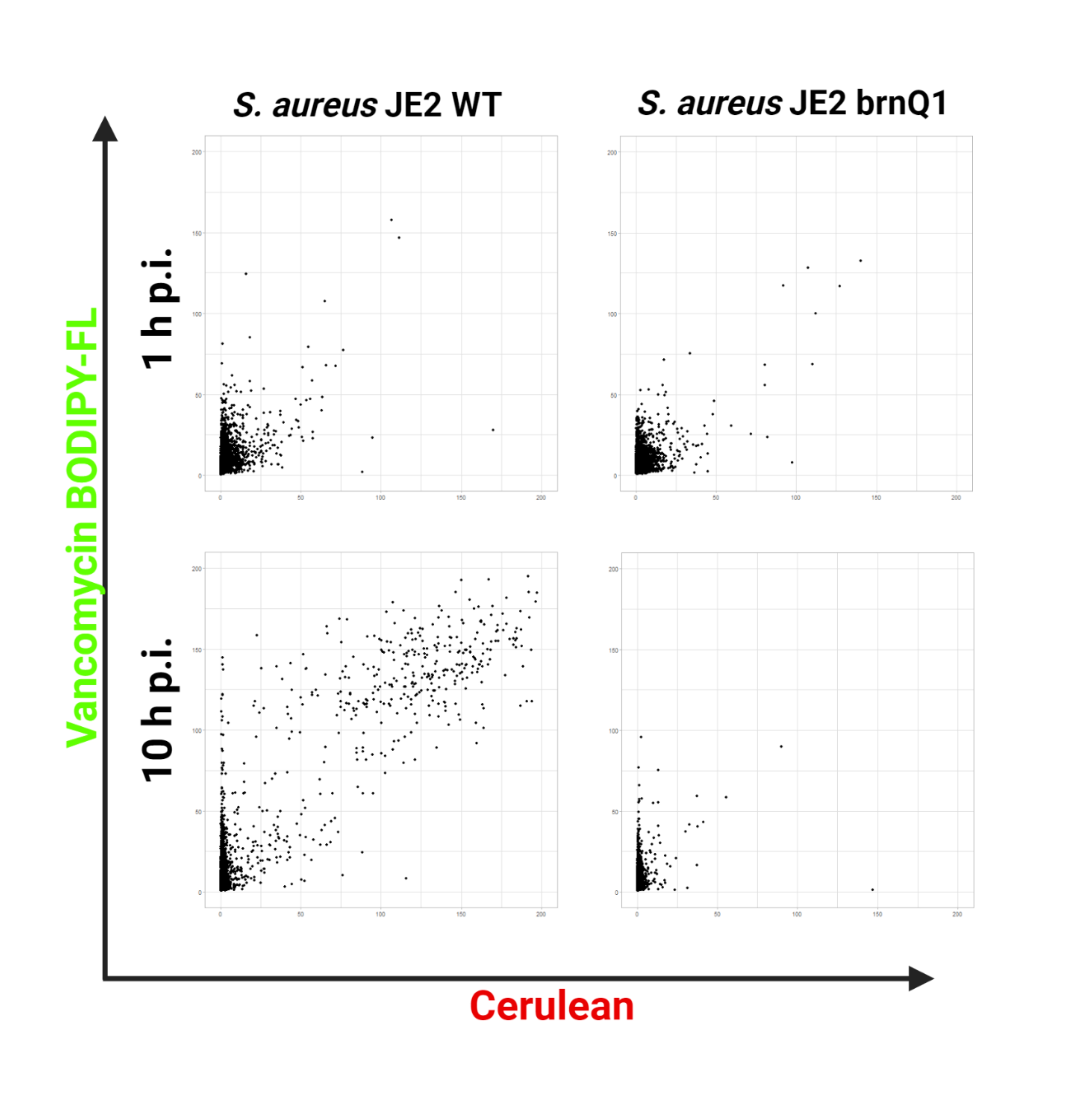

Supplement: S12 Fig — Dot plots detailing the quantification of the percentage of Cerulean-expressing bacteria of all bacteria (labelled with Vancomycin BODIPY FL). Individual bacteria were identified by fluorescence microscopy and were labelled as regions -of-interest (ROI). For each ROI, fluorescence mean intensities in Cerulean and BODIPY-FL were measured and were plotted to visualize Cerulean expression of single bacteria after induction at 1h and 10h p.i. (TIF) [file ppat.1013291.s012.tif]

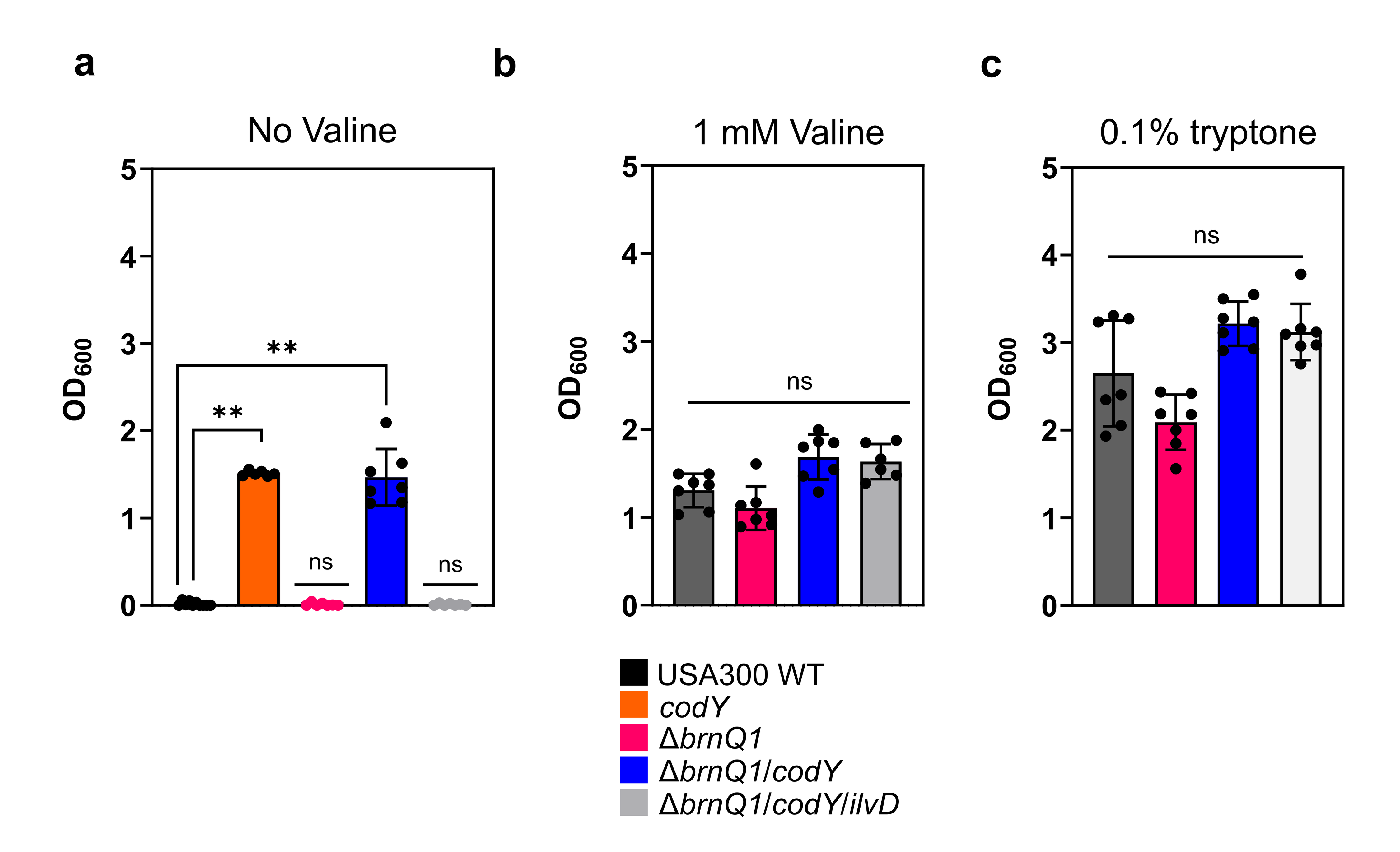

Supplement: S13 Fig — (a) S. aureus JE2 growth in a chemically defined media, either containing 1 mM of each BCAA (CDM) or lacking the stated amino acid(s). OD600 was measured every 18 min, for 48h. Data are shown as mean values ±SD of independent experiments (n = 4). (b) Intracellular growth of a S. aureus brnQ1 in RAW 264.7 macrophages. Macrophages were infected with either WT USA300 or S. aureus brnQ1 and, after gentamicin treatment, supplemented with either valine (Val), leucine (Leu), or Leu and Val (each at 1 mM final concentration) for the duration of the experiment. Data are shown as the mean log10 value ± S.D. for the calculated fold change in CFU/mL at 20h relative to 1.5h p.i., for each bacterial strain. Each data point plotted represents a biological replicate derived from at least three independent experiments (n ≥ 3). Statistical analysis: Brown-Forsythe and Welch ANOVA with Dunnett’s T3 multiple comparison test. (c) Fluorescence microscopy micrographs of RAW 264.7 macrophages infected with S. aureus USA300 WT and S. aureus brnQ1 mutant expressing GFP (green) that were labeled with a fluorescent proliferation dye (blue). The macrophage plasmalemma and extracellular cocci are stained with TMR-WGA (red). 1 mM Val and Leu (each) were added to the medium after gentamicin treatment (1.5h p.i.). At the outset (i.e., 1.5h) all bacteria are GFP and proliferation dye positive; over time and with replication, GFP-positive yet proliferation dye negative bacteria that are devoid of WGA can be seen at 10h and 20h p.i. White arrows indicate unrestricted bacterial replication (URG). Shown are representative micrographs, from 2 independent experiments (n = 2) (scale bars= ~ 10 µm). (TIF) [file ppat.1013291.s013.tif]

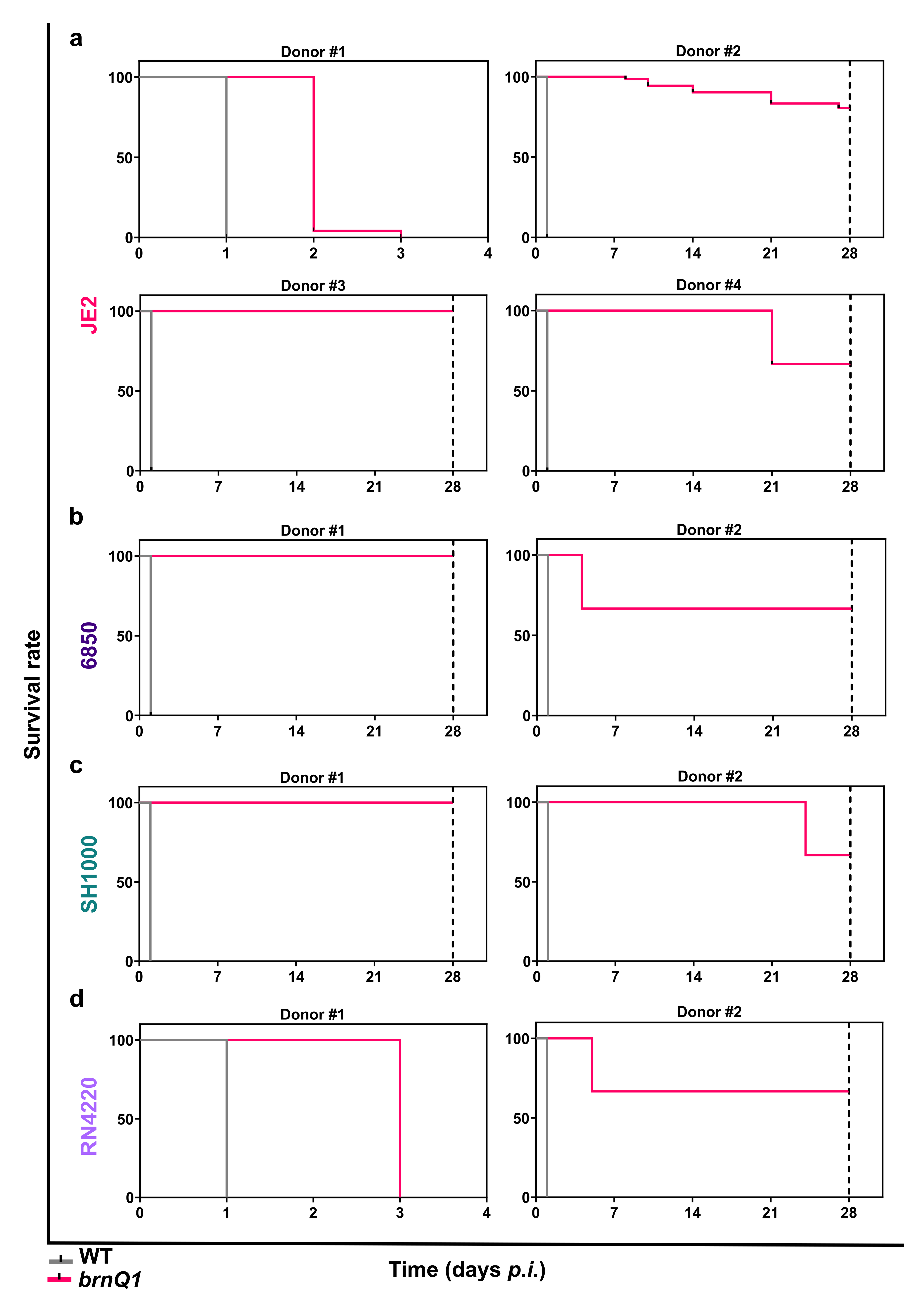

Supplement: S15 Fig — Macrophages were generated from different donors (JE2 brnQ1: n = 4; 6850 brnQ1, SH1000 brnQ1 and RN4220 brnQ1: n = 2) and seeded in well plates as technical replicates (at least 3 wells; JE2 brnQ1 Donor #1: 72 wells; JE2 Donor #2: 80 wells). Infected plates were observed daily and the number of wells, as well as the time point when URG occurred were recorded and plotted in a Kaplan-Meier survival curve. Each well represents in this case a test subject. Wells infected with the parental wild-type strain served as positive controls. (TIF) [file ppat.1013291.s015.tif]

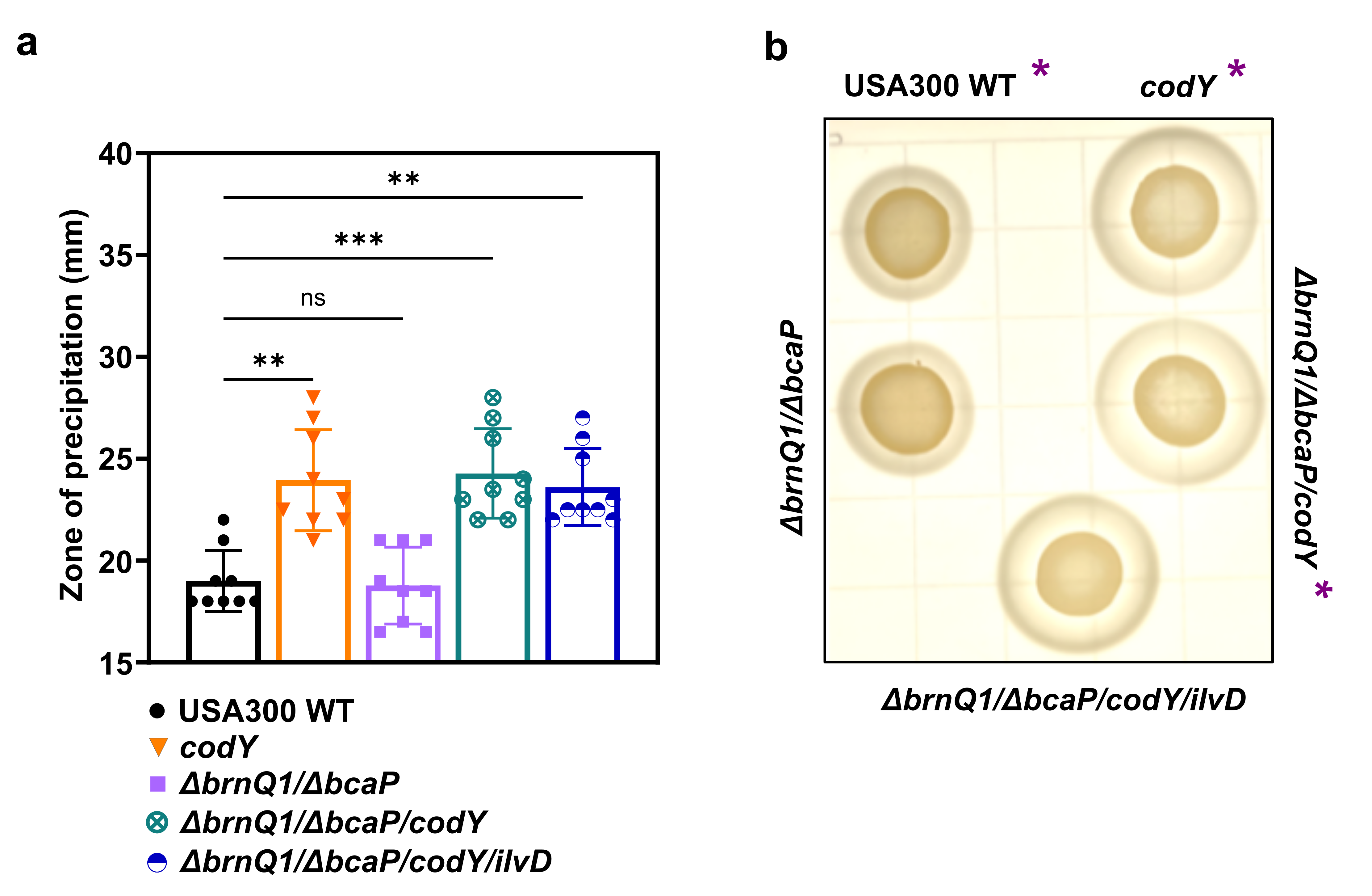

Supplement: S16 Fig — (a) Quantitation of the zones of precipitation around the bacteria grown on SCMA plates for the detection of proteolytic activity shown in (b). Data are shown as mean values ± SD, from independent experiments (n > 3). Statistical analysis: Kruskal-Wallis test with Dunn’s multiple comparisons test; **p < 0.01, ***p < 0.001, ns = not significant. (codY = USA300 codY::Tn; ΔbrnQ1/ΔbcaP = USA300 double deletion mutant of brnQ1 and bcaP; ΔbrnQ1/ΔbcaP/codY = USA300 triple mutant ΔbrnQ1/ΔbcaP/codY::Tn; ΔbrnQ1/ΔbcaP/codY/ilvD = USA300 quadruple mutant ΔbrnQ1/ΔbcaP/codY::Tn/ilvD::Tn-KanR). Asterisks (magenta) designate strains where unrestricted growth (URG) in macrophages was observed (data shown in Fig 1c and Fig 4e). (TIF) [file ppat.1013291.s016.tif]
