# Supplementary material for: Inactivation of branched-chain amino acid uptake halts Staphylococcus aureus growth and induces bacterial quiescence within macrophages
Source: PLoS Pathog. 2025 Aug 8;21(8):e1013291. doi: 10.1371/journal.ppat.1013291 (PMC12333996; doi:10.1371/journal.ppat.1013291)
Supplement: S1 Table — (PDF) [file ppat.1013291.s023.pdf]

Table S1 Bacterial strains used in this study

Strain	Description	Source/Reference
Staphylococcus aureus		
JE2 WT	Plasmid-cured, USA300 LAC derivative, methicillin-resistant (MRSA)	Lab stock, [1]
JE2 WT + mRFP	JE2 WT carrying pSarAP1-mRFPmars; constitutive red-fluorescent protein mRFP expression; Chl^R	This study
JE2 WT + pCerulean	JE2 WT carrying pCerulean; anhydrous-tetracycline inducible cyan-fluorescent protein Cerulean expression; Chl^R	This study
JE2 WT + pP$_{less}$_*brnQ1*	JE2 WT + promoter-less (P$_{less}$) *brnQ1*; backbone control for complementation plasmid; Chl^R	This study
JE2 NE1555 (*codY*) *	JE2 *codY*::*Tn*, deficient in the transcriptional regulator CodY (SAUSA300_1148/SAUSA300_RS06210); Ery^R	[1]
JE2 NE206 (*bcaP*) *	JE2 *bcaP*::*Tn*, deficient in the BCAA transporter BcaP (SAUSA300_2538/ SAUSA300_RS14085); Ery^R	[1]
JE2 NE945 (*brnQ1*) *	JE2 *brnQ1*::*Tn*, deficient in the BCAA transporter BrnQ1 (SAUSA300_0188/ SAUSA300_RS00985); Ery^R	[1]
JE2 NE605 (*brnQ2*) *	JE2 *brnQ2*::*Tn*, deficient in the BrnQ2 isoleucine transporter (SAUSA300_0306/ SAUSA300_RS01630); Ery^R	[1]
JE2 NE44 (*brnQ3*) *	JE2 *brnQ3*::*Tn*, deficient in the putative BCAA transporter BrnQ3 (SAUSA300_1300/SAUSA300_RS07075); Ery^R	[1]
JE2 NE718 (*ilvD*) *	JE2 *ilvD*::*Tn*, deficient in the dihydroxy-acid dehydratase IlvD (SAUSA300_2006/ SAUSA300_RS11035); Ery^R	[1]
JE2 NE292 (*ilvE*) *	JE2 *ilv*E::*Tn*, deficient in the BCAA aminotransferase IlvE (SAUSA300_0539/ SAUSA300_RS02880); Ery^R	[1]
JE2 NE1103 (*leuA*) *	JE2 *leuA*::*Tn* deficient in the 2-isopropylmalate synthase LeuA (SAUSA300_2010/SAUSA300_RS11055), Ery^R	[1]
JE2 *brnQ1*	JE2 transduced with *brnQ1*::*Tn* from the Nebraska Transposon Mutant Library (NE945); Ery^R	This study
JE2 *brnQ1* + mRFP	JE2 *brnQ1* carrying pSarAP1-mRFPmars; constitutive red-fluorescent protein mRFP expression; Ery^R, Chl^R	This study
JE2 *brnQ1* + pCerulean	JE2 *brnQ1* carrying pCerulean; anhydrous-tetracycline inducible cyan-fluorescent protein Cerulean expression; Ery^R, Chl^R	This study
JE2 *brnQ1* + p*brnQ1*	JE2 *brnQ1* + p*brnQ1*; *brnQ1* complementation plasmid (native promoter); Ery^R, Chl^R	This study
JE2 *brnQ1* + pP$_{less}$_*brnQ1*	JE2 *brn*Q1 + promoter-less (P$_{less}$) *brnQ1*; backbone control for complementation plasmid; Ery^R, Chl^R	This study
MJH010 *fur*::tet	*S. aureus* 8325-4 with *tet* cassette inserted in *fur* (SAUPAN004031000)	[2]
JE2 *fur*	JE2 *fur*::*tet* mutant, produced by phage transduction from MJH010 *fur*::*tet*	This study

JE2 *brnQ1/fur*	JE2 *brnQ1* with *tet* cassette inserted in *fur*, produced by phage transduction from MJH010 *fur*::*tet*; Ery^R, Tet^R	This study
Cowan I	NCTC 8530, isolated from septic arthritis, *agr* dysfunction, low expression of toxins and proteases	ATCC 12598, Lab stock
6850	Clinical osteomyelitis isolate, methicillin-sensitive	[3], Lab stock
6850 *brnQ1*	6850 *brnQ1*::*Tn*, *brnQ1* transposon mutant, produced by phage transduction from JE2 *brnQ1*; Ery^R	This study
RN4220	Restriction-deficient derivative of NCTC 8325-4 (cured of prophages Φ11, Φ12, Φ13), β-toxin producer, no production of α-toxin or δ-toxin, phenotypically *agr*-negative	[4], Lab stock
RN4220 *brnQ1*	RN4220 *brnQ1*::*Tn*, *brnQ1* transposon mutant, produced by phage transduction from JE2 *brnQ1*; Ery^R	This study
SH1000	Functional *rsbU* derivative of 8325-4	[5], Lab stock
SH1000 *brnQ1*	SH1000 *brnQ1*::*Tn*, *brnQ1* transposon mutant, produced by phage transduction from JE2 *brnQ1*; Ery^R	This study
USA300	*S. aureus* USA300 LAC cured of its antibiotic resistance plasmid	[6]
Δ*brnQ1*	USA300 carrying an unmarked deletion of the *brnQ1* gene	[7]
Δ*codY*	USA300 transduced with *codY*::*Tn* (NE1555) from the Nebraska Transposon Mutant Library; Ery^R	[8]
Δ*brnQ1/codY*	USA300 carrying an unmarked deletion of the *brnQ1* with *codY*::*Tn* (Ery^R)	This study
Δ*brnQ1/codY/ilvD*	USA300 carrying an unmarked deletion of the *brnQ1* gene with *codY*::*Tn* (Ery^R) and with *ilvD*::*Tn* (Kan^R)	This study
Δ*brnQ1*/Δ*bcaP*	USA300 carrying unmarked deletions of *brnQ1* and *bcaP*	[9]
Δ*brnQ1*/Δ*bcaP/codY*	USA300 carrying unmarked deletions of *brnQ1* and *bcaP* with *codY*:: *Tn* (Ery^R)	This study
Δ*brnQ*/Δ*bcaP/codY/ilvD*	USA300 carrying unmarked deletions of *brnQ1* and *bcaP* with *codY*::*Tn* (Ery^R) and with *ilvD*::*Tn* (Kan^R)	This study
Δ*brnQ1* + pRMC2	USA300 carrying an unmarked deletion of *brnQ1* carrying the empty pRMC2 plasmid; Chl^R	[8]
Δ*brnQ1* + p*bcaP*	USA300 carrying an unmarked deletion of *brnQ1* carrying the pRMC2 plasmid with the *bcaP* gene (p*bcaP*); Chl^R	[8]
Δ*brnQ1* + p*brnQ1*	USA300 carrying an unmarked deletion of *brnQ1* carrying the pRMC2 plasmid with the *brnQ1* gene (p*brnQ1*); Chl^R	[7]
JE2 NE718 (*ilvD)* + pKAN	JE2 *ilvD*::*Tn* transformed with the plasmid pKAN; Ery^R Chl^R	This study
JE2 *ilvD*-Kan	JE2 *ilvD*::*Tn* where the erythromycin resistance casette was replaced with the kanamycin resistance cassette from pKAN; Kan^R	This study
USA300 + pCG44	*S. aureus* USA300 LAC cured of its antibiotic resistance plasmid carrying the plasmid pCG44; Chl^R	[10]

Δ*brnQ1* + pCG44	USA300 carrying an unmarked deletion of the *brnQ1* gene carrying the plasmid pCG44; Chl^R	This study
Escherichia coli		
DH5α	*fhuA2 lac*Δ*U169 phoA glnV44 Φ80' lacZ*ΔM15 *gyrA*96 *recA1 relA1 endA1 thi-1 hsdR17*	BRL Life Technology, Lab stock

* For experiments depicted in Fig. S5b, these strains were transformed with pSarAP1-mRFPmars to allow constitutive red-fluorescent protein mRFP expression; Ery^R, Chl^R (this study).

References

1. Fey, P.D., et al., *A Genetic Resource for Rapid and Comprehensive Phenotype Screening of Nonessential Staphylococcus aureus Genes.* mBio, 2013. **4**(1): p. e00537-12.
2. Horsburgh, M.J., E. Ingham, and S.J. Foster, *In Staphylococcus aureus, fur is an interactive regulator with PerR, contributes to virulence, and Is necessary for oxidative stress resistance through positive regulation of catalase and iron homeostasis.* J Bacteriol, 2001. **183**(2): p. 468-75.
3. Vann, J.M. and R.A. Proctor, *Ingestion of Staphylococcus aureus by bovine endothelial cells results in time- and inoculum-dependent damage to endothelial cell monolayers.* Infect Immun, 1987. **55**(9): p. 2155-63.
4. Kreiswirth, B.N., et al., *The toxic shock syndrome exotoxin structural gene is not detectably transmitted by a prophage.* Nature, 1983. **305**(5936): p. 709-712.
5. Horsburgh, M.J., et al., *sigmaB modulates virulence determinant expression and stress resistance: characterization of a functional rsbU strain derived from Staphylococcus aureus 8325-4.* J Bacteriol, 2002. **184**(19): p. 5457-67.
6. Arsic, B., et al., *Induction of the Staphylococcal Proteolytic Cascade by Antimicrobial Fatty Acids in Community Acquired Methicillin Resistant Staphylococcus aureus.* PLOS ONE, 2012. **7**(9): p. e45952.
7. Kaiser, J.C., et al., *Role of BrnQ1 and BrnQ2 in branched-chain amino acid transport and virulence in Staphylococcus aureus.* Infect Immun, 2015. **83**(3): p. 1019-29.
8. Kaiser, J.C., et al., *The role of two branched-chain amino acid transporters in Staphylococcus aureus growth, membrane fatty acid composition and virulence.* Mol Microbiol, 2016. **102**(5): p. 850-864.
9. Kaiser, J.C., et al., *Repression of branched-chain amino acid synthesis in Staphylococcus aureus is mediated by isoleucine via CodY, and by a leucine-rich attenuator peptide.* PLoS Genet, 2018. **14**(1): p. e1007159.
10. Flannagan, R.S., et al., *Staphylococcus aureus Uses the GraXRS Regulatory System To Sense and Adapt to the Acidified Phagolysosome in Macrophages.* mBio, 2018. **9**(4): p. e01143-18.
