# Supplementary material for: Inactivation of branched-chain amino acid uptake halts Staphylococcus aureus growth and induces bacterial quiescence within macrophages
Source: PLoS Pathog. 2025 Aug 8;21(8):e1013291. doi: 10.1371/journal.ppat.1013291 (PMC12333996; doi:10.1371/journal.ppat.1013291)
Supplement: S2 Table — (PDF) [file ppat.1013291.s024.pdf]

Table S2 - Plasmids used in this study

Plasmid	Description	Reference
p2085	Derivative of pALC2084 [1] with modified multiple-cloning site, shuttle vector, containing *tetR* and P*xyl*/*tet* promoter driving *GFPuvr* expression; Chl^R	[2]
p*brnQ1*	p2085-*brnQ1*, complementation of *brnQ1*, native promotor of *brnQ1*; Chl^R	This study
pP_{less}_*brnQ1*	p2085-*brnQ1*, complementation plasmid vector control; contains *brnQ1* without a promoter region; Chl^R	This study
pSarAP1-mRFP	p2085-SarAP1-mRFPmars, vector expressing mRFP in *S. aureus* under control of the constitutive *sarAP1* promoter; Chl^R	[3]
pCerulean	p2085_Cerulean; expression of cyan-fluorescent protein Cerulean under anhydrous tetracycline inducible promoter; Chl^R	[3]
pRMC2	An anhydrotetracycline-inducible expression vector; Chl^R	[4]
p*bcaP*	pRMC2 plasmid carrying *bcaP* under the control of its native promoter amplified from *S. aureus* USA300; Chl^R	[5]
p*brnQ1*	pRMC2 plasmid carrying *brnQ1* under the control of its native promoter amplified from *S. aureus* USA300; Chl^R	[6]
pKAN	A modified temperature sensitive plasmid to allow for replacement of the Ery^R cassette within Nebraska Transposon Mutants with a kanamycin resistance cassette; Chl^R	[7]
pCG44	A constitutive pHluorin expression plasmid used to visualize *S. aureus* by fluorescent protein expression; Chl^R	[8]

All plasmids contain an Amp^R cassette (*bla*) for plasmid maintenance in *E. coli.*
Antibiotic resistance abbreviations: Amp^R: ampicillin; Chl^R: chloramphenicol; tetracycline: Tet^R; erythromycin: Ery^R; kanamycin: Kan^R.

References

1. Bateman, B.T., et al., *Evaluation of a tetracycline-inducible promoter in Staphylococcus aureus in vitro and in vivo and its application in demonstrating the role of sigB in microcolony formation.* Infect Immun, 2001. **69**(12): p. 7851-7.
2. Giese, B., et al., *Staphylococcal alpha-toxin is not sufficient to mediate escape from phagolysosomes in upper-airway epithelial cells.* Infect Immun, 2009. **77**(9): p. 3611-25.
3. Paprotka, K., B. Giese, and M.J. Fraunholz, *Codon-improved fluorescent proteins in investigation of Staphylococcus aureus host pathogen interactions.* J Microbiol Methods, 2010. **83**(1): p. 82-6.
4. Corrigan, R.M. and T.J. Foster, *An improved tetracycline-inducible expression vector for Staphylococcus aureus.* Plasmid, 2009. **61**(2): p. 126-129.
5. Kaiser, J.C., et al., *The role of two branched-chain amino acid transporters in Staphylococcus aureus growth, membrane fatty acid composition and virulence.* Mol Microbiol, 2016. **102**(5): p. 850-864.
6. Kaiser, J.C., et al., *Role of BrnQ1 and BrnQ2 in branched-chain amino acid transport and virulence in Staphylococcus aureus.* Infect Immun, 2015. **83**(3): p. 1019-29.
7. Bose, J.L., P.D. Fey, and K.W. Bayles, *Genetic Tools To Enhance the Study of Gene Function and Regulation in Staphylococcus aureus.* Applied and Environmental Microbiology, 2013. **79**(7): p. 2218-2224.
8. Gries, C.M., et al., *Potassium Uptake Modulates Staphylococcus aureus Metabolism.* mSphere, 2016. **1**(3).
