# Supplementary material for: Inactivation of branched-chain amino acid uptake halts Staphylococcus aureus growth and induces bacterial quiescence within macrophages
Source: PLoS Pathog. 2025 Aug 8;21(8):e1013291. doi: 10.1371/journal.ppat.1013291 (PMC12333996; doi:10.1371/journal.ppat.1013291)
Supplement: S3 Table — (PDF) [file ppat.1013291.s025.pdf]

Table S3 - Oligonucleotides used in this study

Name	Sequence (5'-3')	Purpose
brnQ1_fwd	GGAGAACAAATACATATATG	*brnQ1* specific primer used to confirm transposon insertion correctness in NE945 and JE2 *brnQ1*
brnQ1_rev	ACGATAAGTGACAACAGG	
brnQ1_Comp_Inf_fwd	AAGGTTATGCTGCTTATTATGCTTGTGGAATTTTACGATG	Amplification of *brnQ1* (native promoter) for cloning p*brnQ1* complementation plasmid
brnQ1_Comp_Inf_rev	CCATGCCATGTGTAAACATATACTTCCCCCCTTCG	
p2085_Inf_for brnQ1_fwd	TTACACATGGCATGGATGAGC	p2085 plasmid amplification for cloning p*brnQ1* complementation plasmid
p2085_Inf_for brnQ1_rev	AAGCAGCATAACCTTTTTCCG	
brnQ1_P-less_Comp_Inf_rev	CCATGCCATGTGTAAAAGAAGAAACTAACATTTAAAGAAA	Amplification of promoter-less *brnQ1* for cloning pP_{less}_*brnQ1*
brnQ1_check1_fwd	TGAAGTTACCATCACGG	Sequencing p*brnQ1* and pP_{less}_*brnQ1*
brnQ1_check1_rev	TGTAGAGCTCATCCATGCC	
fur_region_fwd	CAATAGATTTAGCAGATTGC	Sequencing the *fur* locus (+ its up-and downstream regions) (SAUSA300_1448/SAUSA300_RS07905)
fur_region_rev	ACATGTTGTAGATATGTATGG	
RT_isdA_fwd	GCTACGAACGCAACTAATAATC	RT-qPCR primers for *isdA* (SAUSA300_1029 / SAUSA300_RS05540)
RT_isdA_rev	GTATTCTTTCCAGAATGATGC	
RT_isdB_fwd	TCAAATGGCGAAGCACAAGC	RT-qPCR primers for *isdB* (SAUSA300_1028 / SAUSA300_RS05535)
RT_isdB_rev	GCATTAGCTACTGGTTTAGTTTC	
RT_srtB_fwd	TAAATAAAGACATTGTTGGATGG	RT-qPCR primers for *srtB* (SAUSA300_1034 / SAUSA300_RS05565)
RT_srtB_rev	TACCTTTACGTCGATGTTCTCGC	
RT_sirA_fwd	TGACGCGACAATTAAGTCCGG	RT-qPCR primers for *sirA* (SAUSA300_0117 / SAUSA300_RS00605)
RT_sirA_rev	CCTGTAGGTGCTGTAGAATCATGG	
RT_sbnC_fwd	ATGAATGGGAAGTGGCTCG	RT-qPCR primers for *sbnC* (SAUSA300_0120 / SAUSA300_RS00620)
RT_sbnC_rev	GCTAATGGATGAAATGGACGAT	
RT_sfaC_fwd	TAACATATACGTTATTGCGTACGC	RT-qPCR primers for *sfaC* (*sfnaC*) (SAUSA300_2137 / SAUSA300_RS11770)
RT_sfaC_rev	GCTACTTCATTTATTTGATGTATGG	

RT_fur_fwd	CTAACGCCACAACGCGAAGC	RT-qPCR primers for *fur* (SAUSA300_1448 / SAUSA300_RS07905)
RT_fur_rev	AAATCAAAACGAGCGACGCC	
RT_opp-3A_fwd	GGTTGTGCTAATGACGATGG	RT-qPCR primers for *opp3A* (SAUSA300_0891 / SAUSA300_RS04805)
RT_opp-3A_rev	ACCTTCGAATGTTTGCGCAG	
RT_opp-3F_fwd	ACGAAGTGAGAGCGATTG	RT-qPCR primers for *opp3F* (SAUSA300_0890 / SAUSA300_RS04800)
RT_opp-3F_rev	GAATCTTTTGTATATCAATACCCTC	
RT_RS13380_fwd	TCTTTTTAATGTGTGTGGGC	RT-qPCR for SAUSA300_2417 / SAUSA300_RS13380)
RT_RS13380_rev	CATCGTAAATCCATCATGGC	
RT_gyrB_fwd	CGACTTTGATCTAGCGAAAG	RT-qPCR for *gyrB* (SAUSA300_0005 / SAUSA300_RS00030); reference gene for RT-qPCR.
RT_gyrB_rev	ATAGCCTGCTTCAATTAACG	
