# Supplementary material for: Inactivation of branched-chain amino acid uptake halts Staphylococcus aureus growth and induces bacterial quiescence within macrophages
Source: PLoS Pathog. 2025 Aug 8;21(8):e1013291. doi: 10.1371/journal.ppat.1013291 (PMC12333996; doi:10.1371/journal.ppat.1013291)
Supplement: S1 Text — (PDF) [file ppat.1013291.s026.pdf]

Supporting additional information

This section provides additional information regarding the role of the BrnQ1 transporter in the interaction of *S. aureus* with non-professional phagocytes, such as epithelial cells, and refers to data shown in Fig S4, S5 and Movies S2-5.

After internalization by non-professional phagocytic cells, such as epithelial cells, *S. aureus* initially resides in phagosomes, which is later followed by phagosomal escape and replication within the cytosolic compartment [1, 2]. The main staphylococcal factors which were shown to contribute to phagosomal escape in epithelial cells include: α-type phenol-soluble modulins (PSMα), together with the *agr* system which directly controls PSM production [1], and the AusAB non-ribosomal peptide synthetase [3] which uses the aromatic amino acids phenylalanine and tyrosine, and the branched-chain amino acid (BCAA) valine to generate the cyclic dipeptides phevalin and tyrvalin [4].

To link the *S. aureus* stringent control over BCAA acquisition and biosynthesis to bacterial virulence in epithelial cells, a small-scale screen for phagosomal escape of *S. aureus* mutants in genes associated with BCAA uptake (*brnQ1-3*) and biosynthesis (*ilvE, ilvD* and *leu*) was conducted (Fig S5b). Growth of these mutants in primary human macrophages is shown in Fig 1c. Transgenic HeLa or 16HBE14o- epithelial cell lines stably expressing the fluorescent reporter YFP-CWT (YFP, coupled to the cell wall targeting domain of lysostaphin) were used to analyse phagosomal escape. In this cell lines, as soon as the phagosomal membrane barrier is breached and *S. aureus* is "free" in the cytosol, the fluorophore is recruited to the staphylococcal cell wall, resulting in fluorescent rings visible around the bacteria, which allows quantification of "escaped bacteria" vs "total intracellular bacteria [1, 5] (Movies S2-5).

We here found that inactivation of BrnQ1 resulted in delayed phagosomal escape in both HeLa and the human bronchial epithelial cell line 16HBE14o- (Fig S4b, c). In HeLa cells, JE2 *brnQ1* showed significantly lower phagosomal escape rates at 4 and 6h *p.i.* compared to the JE2 WT (~62% ± 5% and 81% ± 7% respectively). However, after 8h of infection, the phagosomal escape rates were comparable to the wild-type levels. In 16HBE14o-, following a similar dynamic, phagosomal escape rates increase

steadily over time, reaching levels comparable to the wild type by 8h *p.i.* (Fig S5c). Genetic complementation in trans with functional *brnQ1* restored phagosomal escape to wild type levels in both cell lines.

In epithelial cells, bacterial phagosomal escape precedes replication in the host cell cytosolic compartment. Intracellular replication is associated with cytotoxicity and subsequent host cell death [1, 6]. A reduced phagosomal escape rate usually results in delayed bacterial replication, therefore the role of BrnQ1 in intracellular replication and bacterial survival was assessed next. *S. aureus* Cowan I was used as a non-replicative, non-cytotoxic control [7]. While both JE2 WT and the complemented JE2 *brnQ1* strains replicated efficiently in HeLa cells (~12 fold and ~16 fold respectively, by 6h *p.i.*), the *brn*Q1 mutant was significantly delayed in intracellular replication (~2 fold by 6h *p.i.*). During the first 6h *p.i.*, the replication pattern of the JE2 *brnQ1* mutant closely resembles that of the non-replicative Cowan I control strain. However, at 8h *p.i.*, JE2 *brnQ1* does replicate more efficiently than Cowan I, albeit not at comparable rates to JE2 WT (Fig S5d). Consistent with its replication deficit, JE2 *brnQ1* fails to elicit the same cytotoxic effects against HeLa cells as JE2 WT. At 6 and 8h *p.i.*, cytotoxicity upon infection with JE2 *brnQ1* is comparable to the levels measured for the non-cytotoxic strain Cowan I, even though the bacteria have, by this time, translocated to the cytosol and started to replicate. While JE2 WT and the complemented JE2 *brnQ1* strain show ~70% and ~60% respectively, cytotoxicity against HeLa cells at 8h *p.i.*, JE2 *brnQ1* exhibits only 3% cytotoxicity against the host cells (Fig S5e). We observed a similar defective intracellular replication and cytotoxicity of JE2 *brnQ1* in the 16HBE14o-cells.

References

1. Grosz, M., et al., *Cytoplasmic replication of Staphylococcus aureus upon phagosomal escape triggered by phenol-soluble modulin α.* Cell Microbiol, 2014. **16**(4): p. 451-65.
2. Moldovan, A. and M.J. Fraunholz, *In or out: Phagosomal escape of Staphylococcus aureus.* Cellular Microbiology, 2019. **21**(3): p. e12997.
3. Blättner, S., et al., *Staphylococcus aureus Exploits a Non-ribosomal Cyclic Dipeptide to Modulate Survival within Epithelial Cells and Phagocytes.* PLOS Pathogens, 2016. **12**(9): p. e1005857.
4. Moldovan, A., et al., *The AusAB non-ribosomal peptide synthetase of Staphylococcus aureus preferentially generates phevalin in host-mimicking media.* mBio. **0**(0): p. e00845-24.
5. Gründling, A. and O. Schneewind, *Cross-linked peptidoglycan mediates lysostaphin binding to the cell wall envelope of Staphylococcus aureus.* J Bacteriol, 2006. **188**(7): p. 2463-72.
6. Horn, J., et al., *Inside job: Staphylococcus aureus host-pathogen interactions.* International Journal of Medical Microbiology, 2018. **308**(6): p. 607-624.
7. Stelzner, K., et al., *Intracellular Staphylococcus aureus employs the cysteine protease staphopain A to induce host cell death in epithelial cells.* PLOS Pathogens, 2021. **17**(9): p. e1009874.
