# Supplementary material for: Inactivation of branched-chain amino acid uptake halts Staphylococcus aureus growth and induces bacterial quiescence within macrophages
Source: PLoS Pathog. 2025 Aug 8;21(8):e1013291. doi: 10.1371/journal.ppat.1013291 (PMC12333996; doi:10.1371/journal.ppat.1013291)
Supplement: Attachment — Submitted filename: Response to Reviewers.docx [file ppat.1013291.s027.docx]

# Reviewers' Comments:

## Part I - Summary

Please use this section to discuss strengths/weaknesses of study, novelty/significance, general execution and scholarship.

**Reviewer #1:**

The study by Moldovan et al describes the consequences of a mutation in the branched chain amino acid transporter BrnQ1 for intracellular growth and protein synthesis in S. aureus. Interestingly, mutation of codY restores growth, showing that repression of BCAA biosynthesis genes occurs in the macrophage, although the mechanism responsible for this is unexplored. Aside from the key finding (S. aureus uses exogenous BCAAs in the phagosome and requires BrnQ1), the rest of the paper is of more limited significance.

**Reviewer #2:**

This manuscript describes the role of branched-chain amino acids (BCAA) in the survival of S. aureus within phagolysosomes after engulfment by macrophages. The authors analyzed the ability of individual S. aureus mutants deficient for the BCAA transporters BrnQ1, BrnQ2, BrnQ3 and BcaP to replicate and survive within macrophages. They also included mutants lacking the ilvE, ilvD and leuA genes involved in BCAA biosynthesis. Of all these tested, only the brnQ1 mutant was found to be defective in intracellular replication within macrophages. Importantly, this phenotype was observed in multiple S. aureus strains and a growth delay was observed in defined medium with low BCAA availability. The brnQ1 mutant also failed to induce death of the macrophages and remained intracellular, unlike other strains, which ultimately escaped and infected neighboring cells.

Transcriptome analysis revealed the downregulation of genes involved in iron acquisition in the brnQ1 mutant compared to the WT strain, along with several other virulence genes, consistent with studies demonstrating the limited iron availability of a phagolysosome. The results here are clear but without more information **about the rationale for picking 10 hrs post infection** to collect samples for these experiments, it is difficult to completely interpret these results. An interesting aspect of this study was that an iron regulated gene was not expressed in the brnQ1/fur background, suggesting the cells are entering a state of quiescence. This was nicely tested by attempting to induce a reporter gene in the brnQ1 mutant, which demonstrated reduced reporter expression compared with WT cells. In addition, using a combination of mutations that inactivate various genes involved in BCAA regulation, uptake, and/or biosynthesis, the authors conclusively demonstrated that S. aureus could overcome the limited BCAA levels in a phagolysosome by a combination of increased expression of CodY-regulated BCAA transporters or biosynthetic genes. Finally, long-term intracellular survival assays demonstrated the brnQ1 mutant persisted for up to 28 days within a macrophage. These survivors were found to be distinct from classical persisters (SCVs), which exhibit slow growth, antibiotic tolerance, and loss of pigmentation, none of which are characteristics of the brnQ1 mutant cells.

The reason we decide to pick 10h *p.i.* as a key time-point was that, we think, this is the “last moment” both wild-type and the *brnQ1* mutant are still confined intracellularly, with the wild-type already replicating, while the mutant does not. At earlier time-points (up to 6h *p.i.* we don’t observe much difference in infection dynamics between the wild-type and mutant (see Fig. S2 in the revised manuscript), and any time-point later than 10h is “too late”, because extracellular replication can already happen for the wild-type. We thought therefore that the 10h time-point is a good compromise to “catch” differences between the intracellular behavior of wild-type vs mutant.

**Reviewer #3:**

This is an interesting manuscript with new insights into the intracellular lifestyle of S. aureus in macrophages depending on BCAA availability.

The manuscript „Inactivation of branched-chain amino acid uptake halts Staphylococcus aureus growth and induces bacterial quiescence within macrophages” by Moldovan et al. investigates the impact of branched-amino acids and the respective transporters of S. aureus on its intracellular fate in macrophages. To show this the authors used macrophages that they developed from monocytes, which they infected with WT S. aureus and different transporter mutants in different S. aureus backgrounds, the complemented mutants and BCAA-synthesis mutants; they performed growth studies with and without external BCAAs; they performed dual RNAseq of infected macrophages revealing that especially iron-dependent genes were differentially expressed in WT and the mutant indicating that brnQ1-mutants are impaired in gene expression, in de novo protein synthesis representing a quiescent status. However, by external BCAAs supplementation the mutant acted comparable to the WT. The authors investigated the role of CodY for the effects observed in the brnQ1-strain by constructing a brnQ1-codY double mutant, which showed intracellular replication and escape of the phagolysosome. The authors also determined the role of CodY as the regulator of BCAA transporters and synthesis genes including the other important BCAA transporter BcaP and the most important synthesis gene ilvD. The authors also performed longterm infection assays revealing that the mutants could survive during this period. The authors tried to show that the persisting mutants were neither classical persisters nor SCVs.

## Part II – Major Issues: Key Experiments Required for Acceptance

Please use this section to detail the key new experiments or modifications of existing experiments that should be absolutely required to validate study conclusions.

Generally, there should be no more than 3 such required experiments or major modifications for a "Major Revision" recommendation. If more than 3 experiments are necessary to validate the study conclusions, then you are encouraged to recommend "Reject".

Decisive stuff

**Reviewer #1:**

The antibiotic killing experiment is unusual in that it does not include a wild-type strain and the level of killing by rifampicin is not compared with anything. These experiments should be done more completely with the wild-type, mutant and multiple antibiotics, or (as I strongly recommend), removed from the manuscript altogether.

In our experiment, the total duration of RIF treatment was 24h. However, WT *S. aureus* become extracellular during this assayed time. In this case, the effect of RIF, which is present in the medium, on intracellular vs extracellular bacteria can no longer be distinguished. Since it is hard to “maintain” an intracellular population of *S. aureus* WT for 24h, without the addition of other anti-staphylococcal agents (e.g. lysostaphin), which could additionally confound the outcome, we did not include these data in the final manuscript.

The graph below represents a preliminary experiment (n=3), where we infected macrophages (30min), digested extracellular bacteria with lysostaphin (15min), replaced medium with *Infection medium* or *Infection Medium* containing either RIF or DMSO. We counted CFUs at either 6 or 24h *p.i*. Both for wild-type and *brnQ1* mutant, we do not see an effect of RIF at 6h *p.i.* (likely because the RIF treatment is too short to allow proper antibiotic uptake and delivery to the phagolysosome). At 24h *p.i.,* however, we observe a massive difference in CFU recovery of the wild-type treated with RIF vs DMSO. The difference is a result of bacterial unrestricted growth, whenever RIF is not present, making it difficult to assess the effect of RIF on intracellular bacteria. We could not shorten the duration of the RIF treatment itself (24h), because we found, in this case, the antibiotic to not be efficiently taken up by the macrophages.

According to the Reviewer’s suggestion, we thus entirely removed the RIF experiment from the manuscript (Fig. 5h, i, j and Supporting Fig. 14, in the original manuscript).

**Reviewer #2: None**

**Reviewer #3: Major points of criticism**

1. While the authors describe in the introduction the role of S. aureus and also of branched-chain amino acids, it would be interesting for the reader why the authors got the idea that BCAA could play any role for internalized S. aureus.

We knew from previous work [1-3] that BCAAs are important for *S. aureus,* both from growth curve experiments and *in vivo* from murine experiments, therefore we were curious what would happen in macrophages with mutants in these genes. To date, other than the GraXRS system [4], no other staphylococcal factors have been shown to play a role in macrophages. “Usual suspects”, such as toxins, proteases (such as staphopain [5]], the agr system, the sae system, which are important in non-professional phagocytes, did not appear to be relevant during infection of macrophages. Our first surprising finding in macrophages was that *S. aureus* Cowan I and RN4220, which are in general regarded as non-cytotoxic against epithelial cells (among other reasons, due to inactive agr) and often represent our control strains in infection experiments with non-professional phagocytes [5, 6], replicated in macrophages (*unrestricted growth*) (Fig. 1c). We, therefore, thought that mutants in BCAA metabolism might be good candidates as “players” in intracellular infections.

2. Also, is this an observation which holds only true for professional phagocytes or is this also a mechanism for intracellular survival in non-professional phagocytes such as epithelial or endothelial cells?

The dynamics of *S. aureus* are different in macrophages vs epithelial cells. In macrophages, bacteria start to replicate directly within phagosomes. In epithelial cells, *S. aureus* has to first escape phagosomal enclosure, to be able to replicate in the cytosol. In epithelial cells, bacteria which can’t escape or have very reduced phagosomal escape rates. (e.g. *S. aureus* Cowan I, RN4220, *S. aureus* Δ*agr, S. aureus* Δ*psmα*) also do not replicate intracellularly [6-9].

We have now included data in the revised manuscript describing the involvement of BrnQ1 in the interaction with epithelial cells (HeLa and the human bronchial epithelial cells 16HBE14o-). The information can be found in supporting Fig. S4, S5, Supporting Information and supporting Movies S2-5.

In case of the *brnQ1* mutant (but not for mutants in any of the mutants screened in Fig. 1c (*brnQ2, brnQ3, ilvE, ilvD, leuA*) we observed a delay in phagosomal escape (Fig. S5 b-c), accompanied by a delayed replication (Fig. S5 d) and reduced cytotoxic effects against host (Fig. S5 e). We observe these effects both in HeLa and HBE cells (although, in general, HeLa cells are more susceptible/sensitive to *S. aureus* than HBE).

3. Since the authors do not only use BCAA transporter mutants, but also mutants in genes for BCAA synthesis, they should introduce the synthesis genes in the introduction. Furthermore, is it known if there is any phenotype associated with the single BCAA synthesis mutants? Can this be expected, since mostly S. aureus uses BCAAs from the environment as it is described in several studies (e.g. Kaiser et al. PlosGen2018), where it also has been described that it has been suggested that S. aureus would be auxotrophic for leucine and valine.

We included in the *Introduction* statements about BCAA biosynthesis as well as the role of the CodY repressor in the control of BCAA metabolism.

In accordance with the observations regarding the hierarchy of CodY-elicited control of *S. aureus* gene regulation [10] [also reported in *B. subtilis [**11**]*], upon nutrient-depletion (especially Ile and GTP), genes controlled by CodY will be gradually de-repressed, as follows:

**Cluster 1**: includes genes which encode BCAA, histidine and methionine transporters (all are very sensitive to minimal drops in Ile concentrations)

**Cluster 2**: includes genes involved in purine biosynthesis (eg. *purCSQLFMHD*)

**Cluster 3**: includes genes involved in heme-based iron acquisition (eg *isdCDE*), genes of the *agr* RNA II transcript (*agrBDCA)*, most of the BCAA biosynthetic cluster (*ilvDBNC, leuABCD, ilvA*)

**Cluster 4**: includes the *hld* delta toxin (*agr* RNA III transcript), the *ilvE* gene (part of the BCAA biosynthetic machinery, but not part of the *ilv-leu* operon), capsule biogenesis genes (*cap* operon).

**Cluster 5**: includes some genes which are positively regulated by CodY (i.e. their expression drops upon CodY inactivation): genes of the accessory Sec locus or enterotoxin-like protein encoding genes.

Regarding the phenotype of single BCAA synthesis mutants, there is not much known. Under our experimental infection conditions, in either macrophages (Fig. 1c) or epithelial cells (new Fig. S5 b) there seems to be “no need” for BCAA biosynthesis. BCAA biosynthesis seems to be “required” for *S. aureus* infection of macrophages only if both *brnQ1* **AND** *bcaP* are missing (i.e. import of Val and Leu is halted; Ile can still be imported in this case, via BrnQ2). Even upon additional deletion of *codY* a mutant lacking *brnQ1+ bcaP + ilvD* cannot grow intracellularly (despite having the entire “array” of CodY-de-repressed factors at “its disposal”) (Fig. 4e). This highlights the importance/priority of “import” vs “biosynthesis” of BCAAs under conditions with low BCAA availability.

One could speculate that *S. aureus* has adapted to have many mechanisms in place to avoid reaching a point where BCAA biosynthesis is truly needed. The fact that *S. aureus* encodes a dedicated Ile transporter (BrnQ2) suggests that *S. aureus “*tries” to maintain CodY-mediated repression, if possible. *codY* mutants, as well as *brnQ2* mutants, are reported to be hyper-proliferative and hyper-virulent, a state which might not necessarily provide a lot of advantage for long-term survival within a host.

4. Does it really make sense to test a codY mutant in this regard? Since codY is a negative regulator of the BCAA-synthesis genes, in a codY mutant, these genes would be repressed, thereby acting if at all more in a BCAA synthesis way. Therefore, what should be expected in terms of determining the role of the BCAA-synthesis operon?

We apologize if this was confusing. We only included the *codY* mutant as a control. *codY* mutants are known to be hypervirulent[12], precisely because many virulence-relevant genes are de-repressed, together with amino acid and oligopeptide uptake proteins and amino acid biosynthetic genes. Therefore, we expected this strain to not exhibit an impaired growth in macrophages. We have now clarified in the text that the *codY* mutant rather serves as control.

5. The term of “unrestricted growth” after escape from the macrophage, is this a term used in the scientific community or is it a term only used in this study? The authors should describe the effect of this more intensely.

We introduced this term in our study because we felt that it described very well the phenomenon whereby bacteria exit the infected host cell, replicate massively in the extracellular milieu, kill the host cells and end up forming micro-colonies within the infected wells of the cell culture plates and/or biofilms within the wells. Some similar findings were also reported by another study [13].

Because the high number of CFUs recovered at 24h *p.i.* (~ 10, 000 x compared to T0) does not stem from actual intracellular replication, we used the term *unrestricted.* We have altered the text in the *Results* section to clarify what we mean.

6. By first mentioning the Nebraska Transposon Library, the authors should state in which background the library has been established, USA300 LAC.

We included this in the corresponding *Results* section, as well as in the legend for Fig. 1c.

7. Why did the authors use JE2 as another strain for introducing the brnQ1 mutation as indicated line 124? And surprisingly, in line 133 the authors report that the mutation was also moved into the S. aureus strains 6850, SH1000 and RN4220. They should have indicated this earlier.

The “mini-screen” shown in Fig. 1c included mutants retrieved directly from the Nebraska library, which we store in our strain collection directly as it was obtained. We always use these mutants “as such” for any type of screening work.

Whenever we want to conduct more in-depth work with certain mutants, we transduce the mutation from the Nebraska Library strain (USA300 JE2, i.e. USA300 LAC, cured of plasmids *p01* and *p03*) into the *S. aureus* USA300 JE2 strain. It is basically the same strain, but it is the particular clone our lab is using, which has been fully re-sequenced and kept in collection without excessive passaging. We do this step to ensure that we have a true parental wild-type strain to work with and to avoid any unexpected off-target mutation which might be present in the “original” Nebraska Library strain(s). Since we often additionally modify these mutants (e.g. by bringing in additional mutations, complementation plasmids, plasmids carrying fluorescent proteins, etc.), we want to ensure that all the strains which we compare have the same genetic background.

The additional strains we introduce in Fig. 1e (and later in new Fig. S15), 6850, SH100, RN4220 were only added to emphasize that the growth defect we see in the *brnQ1* mutant is not USA300/JE2-specific, but also present in other strains, including the less-virulent RN4220.

We have now tried to better clarify in the text which mutants were used were. We have additionally modified Fig. 1c where we re-labelled the mutant which are directly retrieved from the NTML without further transduction in “our own” parental strain, using the “NE” identifier, to distinguish them from the “clean” mutants we have generated in our parental strain(s).

8. The authors show that the brnQ1 mutation does not only effect intracellular replication but also growth in cell culture and infection media. This is not entirely new as Kaiser et al have already shown in 2015 that this mutant has a growth defect in chemical defined medium without BCAAs (Kaiser et al in 2015, Inf Immun).

Indeed, we are aware that the growth impairment of *brnQ1* mutants under conditions of limited BCAA availability is not a new finding, that is why these data are in the Supporting information and not in the main manuscript.

Nonetheless, we wanted to re-iterate this information here. We found it was especially important to show that *brnQ1* mutants can grow (even if less efficiently than the wild-type) in the media used during the infection experiments to emphasize that the real limitation for this mutant comes when intracellular. These particular media (RPMI1640 or RPMI1640 + FBS were not tested in the [Kaiser et al 2015] study.

9. The paragraph showing that these mutants are not “classical persisters” is not very convincing. The authors do not show data of the real persisters after 28d, but point at the transcriptomic data at 10h p.i. Also, for the antibiotic tolerance, they test infection after 10h. It would be more convincing to test antibiotic tolerance after long-term infection. However, convincing is the description that the mutants do not undergo the SCV status during persistence. A last point of interest would be to search the publicly available S. aureus genome data bases to check if there are any natural brnQ1 existing mutants in clinical isolates present. This would add a clinical role for the interesting mutant.

As per the request of Reviewer 1, we have already removed the RIF data entirely, since our experimental set-up might not have been ideal (see above).

As to the timing of the RNA-seq data: We chose the 10h time-point for RNA-seq, because that was the time-point where we already observed that *brnQ1* mutants have become “unresponsive” to environmental cues, such as iron-deprivation or aTC-induction of the *Cerulean* fluorescent protein (Fig.3).

As to the genome database searches: When we became aware of the major impact of a *brnQ1* mutation during *S. aureus* infections at the beginning of the project, we performed a database search using ~14,000 available whole genome shotguns within GenBank. Nucleotide regions encoding *brnQ1* were identified with BLASTN, the corresponding sequences were subsequently extracted and aligned using ClustalO.

However, we did not detect major mutations within the ORF (aside from incomplete contigs and other sequencing associated errors) suggesting that either *brnQ1* mutants are eliminated *in vivo* or do not contribute to acute virulence so that they are not prevalent in the clinics and thus not analyzed by sequencing efforts.

We generated a sequence alignment and deposited it on the data sharing platform Zenodo (DOI 10.5281/zenodo.15425437; https://zenodo.org/records/15425437). This rather large alignment should be opened with SeaView (https://doua.prabi.fr/software/seaview), since other programs might run into problems due to the size of the dataset.

However, since there does not seem to be a selection for *brnQ1* mutants we did not include this information in the manuscript.

## Part III – Minor Issues: Editorial and Data Presentation Modifications

Please use this section for editorial suggestions as well as relatively minor modifications of existing data that would enhance clarity.

**Reviewer #1:**

The long term survival of the mutant is discussed as though it could represent a long-term survival niche, but the mutant doesn't appear to occur naturally, so this behavior is artifactual and of limited significance. The authors should temper their language when discussing this part of the paper. The comparison with SCVs is of limited relevance as they are clearly completely different things and one would not expect them to be similar. A SCV is generally a S. aureus strain that has lost the ability to respire, while this mutant has lost the ability to take up exogenous BCAAs. Comparison of the two seems unusual. It is recommended that this is reduced or removed or at least justified more thoroughly.

So far, natural mutants within *brnQ1* were indeed not reported - at least judging by the absence of mutations in WGS databases (see our response above: Reviewer 3; point 9.). This does not necessarily mean that they don’t exist- *brnQ1* mutants could be eliminated *in vivo* or not contribute to acute virulence, therefore they are not prevalent in the clinics and thus not analyzed by sequencing efforts.

Usually, SCV formation is selected for during passage of *S. aureus* through the host. SCVs are slowly replicating, non-cytotoxic, non-pigmented variants of the original infection strain. The phenotype is indeed associated with deficiencies in respiratory metabolism.

While the main “feature” of the *brnQ1* mutant is its incapacity to take up BCAAs, one cannot exclude that this halted growth & unresponsive state could result in other downstream defects in this bacterium (e.g. metabolic changes, etc) thus resulting in a SCV-resembling phenotype. Since SCVs are known to be slow growing, we thought that discussing this feature in the case of the *brnQ1* mutant would make sense nonetheless.

While the long-term infection behaviour is artefactual, given that it is a phenotype that we only observed under experimental conditions, to our knowledge, such a phenotype has not yet been reported (i.e. 28 days of infection without harming the host cells, while having no antibiotics/lysostaphin in the culture medium) and might be of importance when studying the intracellular behaviour of *S. aureus.*

In lines 135-145 the authors discuss different media and their BCAA content but they only give actual concentrations of BCAAs for some conditions and are vague for others. Please add quantities for each condition if they are so relevant.

The only conditions where we discuss BCAA content are CDM media (where we indicate the BCAA content: 1 mM each) or the commercially available RPMI1640 (where we indicate the Val and Leu concentrations; we included now the Ile concentration as well). We are not trying to make any specific point here, we just mentioned the numbers simply to illustrate that even with relatively high BCAA availability in our CDM (1 mM), *brnQ1* mutants still have a growth deficit in the absence of peptides & whatever else might be present in a rich, undefined medium such as TSB. We unfortunately do not know what BCAA concentrations might be achieved in the *Infection Medium* (RPMI1640 + 10% v/v FBS) as a result of FBS supplementation. We insisted on including these comparisons to highlight the different behaviors the *brnQ1* mutant exhibits under different environmental conditions:

- no defect in a “rich” culture medium (TSB);
- mild but consistent growth delay if only free amino acids are available (1 mM in CDM; ~130 µM in RPMI1640), even upon FBS supplementation (*Infection Medium*);
- halted growth when intracellular in macrophages (cultivated in *Infection Medium*).

In line 236, recommend removing "Remarkably" as there is no reason to suspect the Fur repressor would be involved in the phenotype.

We removed it from the text.

The reason why we found our observation striking is because *isd* genes are supposed to be completely de-repressed in mutant strain lacking the Fur repressor, regardless of whether BrnQ is active or not.

The macrophage phagosome is a low-iron environment, therefore the *isdB* gene should be de-repressed in WT bacteria (which is exactly what we observe). In a *fur* mutant, the *isdB* gene is constitutively de-repressed. However, the *brnQ1* mutant did not de-repress the *isdB* gene, despite the low-iron conditions. We nonetheless assumed, that whatever the reasons for *brnQ1* mutants to not upregulate *isdB* gene during iron-depletion, this particular phenotype should be rescued if the repressor itself is missing. The fact that mutating fur in the *brnQ1* mutant did not lead to de-repression of *isdB* indicated that these bacteria are actually “non-responsive” to environmental cues.

Fig. 5e and f should be in supplement.

We have made some adjustments to Fig. 5, including the removal of the RIF experiment-specific schematic & graph (Fig. 5h, i and j), as the reviewer has suggested.

We would like, however, to keep Fig.5e and f in the main figure. They show that *brnQ1* clones which grew unrestrictedly from macrophages, did not do so due to some heritable mutations they have acquired during the prolonged stay in the macrophage, as has been previously shown for bacteria grown under Val deprivation, for example [3]. These bacteria retain the *brnQ1* mutant-specific growth defect both during growth in *Infection Medium* and during infection of macrophages.

**Reviewer #2:**

Overall, this is a nicely presented body of work that provides important new insight into the survival and growth of S. aureus after engulfment by macrophages. Although this a very thorough study that addresses numerous aspects of growth within a phagolysosome, my one concern is the characterization of the phenotype observed as “dormancy”, akin to persisters. I agree that it is tempting to speculate that this is a new and previously uncharacterized metabolic state, but without any evidence that this state arises in the WT strain or during an infection, I believe any speculation as to the functional relevance of this state should be made with caution.

Questions and comments:

Line 42: Change, “It is facultative intracellular…”, to “It is a facultative intracellular pathogen…”.

Since we used the word “pathogen” at the beginning of the sentence (*“Staphylococcus aureus is a human pathogen…”* we wanted to avoid the repetition. We have altered the sentence to: *“It is facultatively intracellular and can reside…”.* We hope the Reviewer finds this phrasing suitable.

Line 256: In this context, what does deplete mean? Clearly there is enough BCAAs for growth, as long as the bacteria can transport them in.

We meant “reduced” or “not optimal”, but perhaps “deplete” is an unfortunate term in this case. We have replaced it with “scarce in BCAAs”.

Line 263: I don’t understand this statement: “During growth in CDM lacking all three BCAAs, the growth of S. aureus is preceded by an extended lag phase of ~20h. Omission of Val and Leu, however, completely halted bacterial growth.” If the absence of all three BCAAs causes a lag in growth, why would the absence of only two “completely halt growth”? Please clarify.

We agree that this observation is counterintuitive, we didn’t expect to see this, and we do not have a clear explanation for this. We state this now in line 276 (revised manuscript) that this observation was striking. We did not include further in depth explanation. However, should the reviewer consider more explanations are needed in the main text, we are happy to include details.

So far, the “behaviour” of *S. aureus* during growth in the absence of BCAAs is as follows:

**Complete CDM**: the *ilv-leu* operon is repressed, in an Ile-dependent manner (via CodY) and a Leu-dependent-manner via a Leu-rich attenuator peptide (encoded upstream from *ilvD*, only if Leu is available; prevents transcription of the *ilv* operon by allowing formation of a terminator).

Growth phenotype: normal

**CDM [-Ile]:** de-repression of the *ilv-leu* operon, due to loss of CodY repression.

Growth phenotype: normal or faster.

**CDM [-Val]**: CodY is still active (because there is Ile in the medium); the attenuator peptide is also formed (because there is Leu in the medium) 🡪 *S. aureus* has “no other choice” than to select for suppressor mutants in either *codY* or 5’-UTR of *ilvD.*

Growth phenotype: strongly attenuated (long lag-phase: 20-24h), followed by “normal” growth afterwards.

**CDM [-Leu]**: attenuator peptide-dependent repression is relieved; but CodY is still active.

Growth phenotype: reduced growth rate (i.e. ~6h lag-phase).

**CDM [-Leu] and [-Ile]**: absence of Ile, leads to de-repression of CodY targets *ilv-leu*

Growth phenotype: normal or faster (as expected).

**CDM [-Val] and [-Ile]:** loss of CodY repression of *ilv-leu* BUT:

Growth phenotype: expected: normal; observed: long lag-phase, similar to CDM [-Val]; we would have expected that omission of “Ile” would have rescued the growth defect.

**CDM[-BCAA)**: loss of CodY repression of *ilv-leu* (because Ile is missing); “Loss” of attenuator peptide because Leu is missing;

Growth phenotype: expected: normal; observed: attenuated (~16h lag phase)

Reason: unknown, despite the *ilvD* operon being “ON” (shown by promoter assays); it is possible that the growth impairment could also results from the activity of the biosynthetic enzymes themselves: e.g. the aminotransferase has substrate bias towards Ile or Leu synthesis, or there is negative or positive cross-regulation between the pathways.

**CDM [-Leu] and [-Val]**: CodY repression is maintained (because Ile is still present); the attenuator peptide is not formed (due to Leu missing); due to Val missing, “eventually” suppressor mutants should, theoretically, be selected and allow bacteria to grow.

Growth phenotype: expected: reduced, but shorter lag-phase than in CDM[-Val]; observed: growth is completely halted during the assayed time (60h).

One aspect we have not analysed here, is the general “metabolic” status of the bacteria under these conditions. While the *ilv-leu* operon might be, at least partially, de-repressed (by loss of the Leu-rich attenuator peptide), this does not guarantee that enzyme activity is not affected. Similarly, the initial steps in the BCAA synthesis require threonine (for IlvA) and pyruvate (for IlvN). Should the bacteria be “starved” for too long (i.e. by a very long lag phase, due to the absence of Val), it is possible that enzyme substrates are no longer available.

**Reviewer #3: Minor points of criticism**

Line 58: The description “commensal” should be avoided, see the viewpoint article by Proctor, RA: Clin Infect Dis 2021 Jul 1;73(1):e267-e269. doi: 10.1093/cid/ciaa1431.

Have We Outlived the Concept of Commensalism for Staphylococcus aureus?

Thank you for pointing this out and referring us to this article. We have removed “commensal” and further refer to *S. aureus* as *opportunistic pathogen.*

A legend to Table 1 is missing.

We thank the reviewer for pointing that out! We have added the legend.

Figure 1a: This figure can be deleted, the assay is well described and not especially new.

We agree that there is indeed no particular novelty to our method. However, we would like to keep the schematic. There are as many variations to this infection protocol as infection studies with *S. aureus,* and we wanted to be clear to our readership which method is exactly being used (especially after we “move” to the long-term infection where we really wanted to highlight that no antibiotics were kept in the medium during those 28 days).

Fig. 1f: What do the authors mean by “stills”?

We used to word “stills” to refer to individual frames from time-lapse imaging. But maybe it was an unfortunate use of the term. We replaced “stills” with “micrographs”.

Fig. 1g: In the legend of this figure there is no mentioning of the mutant. What do the authors intend to show here?

We here only wanted to show (qualitative assessment) by TEM, that both wild-type and mutant are confined inside phagosomes at 10h p.i. A wider “picture” of the infection dynamics is more evident in Fig. 1f and Movie S1.

We thus simply used the term “JE2” to state that both JE2 WT and the JE2 *brnQ1* mutant are depicted in the respective panel.

Fig. 3c can be deleted, see commentary to Fig. 1a

Same as mentioned for Fig.1a: although we agree that the schematic doesn’t necessarily show a “groundbreaking” new method, we would again like to offer visual support to the reader when interpreting the assay (i.e. when exactly ATc was added, etc.)

Reference list: In the reference list, all bacteria and gene names should be written in italics. Also, the references should be in a homogenous style with either all journals abbreviated or not.

We used the “*Vancouver”* style for our references, as required by PLOS formatting requirements (<https://journals.plos.org/plospathogens/s/submission-guidelines>). This particular style seems to indeed not italicize either species or gene names. We checked the references for several recently published PLOS Pathogens research articles, and they all show this feature (i.e. no italics for either species or genus).

We assume that the manuscript will have to be anyway re-formatted to 100% comply with PLOS requirements.

We have, however, removed “strange characters” such as “ <i> … <i>”. We have formatted the references all the *Supporting Material* appropriately (i.e. italics for species and gene names).

Lines 257: The authors suggest that they would test the effects of BCAAs to intracellular bacterial growth directly, which is fine. However, next the report at first the results of the effect on isdB expression. It would be more logic to show at first the results of the effects of external BCAAs on growth and then on isdB expression.

We exchanged the graphs and modified the text: we now show bacterial growth first (Fig.3 h-i), then the *isdB* expression (Fig.3j).

Supporting Fig. 12: The authors should also test the effects of “no valine” on a single codY-mutants since the growth of the WT is also affected in this medium.

This is true, we should have included the strain in the graph. We have now modified the figure and corresponding legend to also include the *codY* mutant in the “no valine” condition (new Fig. S14).

**Materials and Methods**

Line 617: Usually, the Thoma counting chamber is used to count eukaryotic cells. How should this work to count bacteria? This should be explained in more detail.

For counting the host cells, we use the improved Neubauer chamber.

The Thoma chamber is, indeed, rather used for counting thrombocytes and erythrocytes. We found, however, that it is also very suitable for counting bacteria.

Bacterial suspensions are prepared for infection and 10 µL is loaded onto the Thoma chamber (see figure below; 20x magnification). Bacteria are allowed to “settle” for ~ 10 min and are afterwards manually counted under a phase contrast microscope, with 20x magnification. The no. of bacteria / mL is estimated using the formula below and used for infection at the desired MOI.

The formula below applies for Thoma chambers with an aria of the small square (yellow) of 0.05 x 0.05 mm^2^ and a depth of 0.1 mm.

We have now included this explanatory note in the Materials & Methods.

$\frac{Total number of bact. counted in "x" yellow squares}{x}\times4x{10}^{6}=bacteria/mL$

Thoma counting chamber with S. aureus suspension (20x)

We have also included a comparison between the bacterial counts get with the Thoma chamber, in comparison to a CFU assay for the same suspension.

In the graph below, we did the following:

1. Counted the bacteria using the Thoma chamber;
2. Based on that, we prepared a bacterial suspension containing the amount of bacteria/mL that we needed for our infection experiment (usually, for MOI5 using 10^5^ seeded macrophages: 5 x 10^5^ bacteria/mL)
3. We used this suspension(s) for infection
4. Also: counted this suspension by CFU assay
5. Divided the number of bacteria that we expect to have in our suspension, based on our Thoma count (“Should”) to the number of bacteria that we actually counted by the CFU assay (“Is”) 🡪 a ratio of ~ 1 indicated that our Thoma chamber-based estimation is accurate enough for our purposes.

Thoma chamber vs CFU counting of bacteria

Line 626: This sentence should be corrected “where” to “with”? Also, “mins” should be corrected to “min”.

Done (we changed “where” to “in”).

Also, the authors should use the same abbreviations throughout the manuscript: hours or h with or without a free space.

Thank you for pointing it out! We tried to eliminate these inconsistencies.

References

1.	Kaiser, J.C., et al., *Role of BrnQ1 and BrnQ2 in branched-chain amino acid transport and virulence in Staphylococcus aureus.* Infect Immun, 2015. **83**(3): p. 1019-29.

2.	Kaiser, J.C., et al., *The role of two branched-chain amino acid transporters in Staphylococcus aureus growth, membrane fatty acid composition and virulence.* Mol Microbiol, 2016. **102**(5): p. 850-864.

3.	Kaiser, J.C., et al., *Repression of branched-chain amino acid synthesis in Staphylococcus aureus is mediated by isoleucine via CodY, and by a leucine-rich attenuator peptide.* PLoS Genet, 2018. **14**(1): p. e1007159.

4.	Flannagan, R.S., et al., *Staphylococcus aureus Uses the GraXRS Regulatory System To Sense and Adapt to the Acidified Phagolysosome in Macrophages.* mBio, 2018. **9**(4): p. e01143-18.

5.	Stelzner, K., et al., *Intracellular Staphylococcus aureus employs the cysteine protease staphopain A to induce host cell death in epithelial cells.* PLOS Pathogens, 2021. **17**(9): p. e1009874.

6.	Grosz, M., et al., *Cytoplasmic replication of Staphylococcus aureus upon phagosomal escape triggered by phenol-soluble modulin α.* Cell Microbiol, 2014. **16**(4): p. 451-65.

7.	Blättner, S., et al., *Staphylococcus aureus Exploits a Non-ribosomal Cyclic Dipeptide to Modulate Survival within Epithelial Cells and Phagocytes.* PLOS Pathogens, 2016. **12**(9): p. e1005857.

8.	Horn, J., et al., *Inside job: Staphylococcus aureus host-pathogen interactions.* International Journal of Medical Microbiology, 2018. **308**(6): p. 607-624.

9.	Moldovan, A. and M.J. Fraunholz, *In or out: Phagosomal escape of Staphylococcus aureus.* Cellular Microbiology, 2019. **21**(3): p. e12997.

10.	Waters, N.R., et al., *A spectrum of CodY activities drives metabolic reorganization and virulence gene expression in Staphylococcus aureus.* Molecular Microbiology, 2016. **101**(3): p. 495-514.

11.	Brinsmade, S.R., et al., *Hierarchical expression of genes controlled by the Bacillus subtilis global regulatory protein CodY.* Proc Natl Acad Sci U S A, 2014. **111**(22): p. 8227-32.

12.	Montgomery, C.P., et al., *CodY Deletion Enhances In Vivo Virulence of Community-Associated Methicillin-Resistant Staphylococcus aureus Clone USA300.* Infection and Immunity, 2012. **80**(7): p. 2382-2389.

13.	Pidwill, G.R., et al., *Clonal population expansion of Staphylococcus aureus occurs due to escape from a finite number of intraphagocyte niches.* Scientific Reports, 2023. **13**(1): p. 1188.
